# BCL2 Facilitates Naïve Pluripotency and an 8C‐Like State in Human Embryonic Stem Cells

**DOI:** 10.1111/cpr.70297

**Published:** 2026-11-08

**Authors:** Zhimin Lu, Han Wu, Xiaomin Su, Hua Yu, Fang Yang, Siqin Bao, Xihe Li, Baojiang Wu

**Affiliations:** ^1^ College of Life Sciences, Research Center for Animal Genetic Resources of Mongolia Plateau Inner Mongolia University Hohhot China; ^2^ International Mongolian Hospital of Inner Mongolia Hohhot China; ^3^ Inner Mongolia Saikexing Institute of Breeding and Reproductive Biotechnology in Domestic Animal Hohhot China

**Keywords:** 8C‐like cells, BCL2, embryonic stem cells, epigenetic, pluripotency, zygotic genome activation

## Abstract

Human naïve embryonic stem cells (ESCs) can spontaneously transition into 8‐cell‐like cells (8CLCs), a cell state that recapitulates the totipotent features of the 8‐cell‐stage embryo. However, the regulatory circuitry underlying this reprogramming process remains poorly elucidated. Here, we show that BCL2 contributes to 8CLC induction. We demonstrate that BCL2 not only stabilizes the human naïve pluripotent state via H3K27ac associated epigenetic modulation but also induces and sustains 8CLC self‐renewal under chemically defined culture conditions. Mechanistically, BCL2 safeguards the expression of core pluripotency genes, endowing cells with self‐renewal capacity and lineage‐biased differentiation capacity, enabling their contribution to both embryonic and extra‐embryonic lineages in chimeric embryos. Notably, BCL2 primes human ESCs to gain competitive dominance in the mouse ESC niche, suppresses mesodermal lineage commitment, and enhances trophoblast differentiation. Taken together, our findings reveal a role for BCL2 in regulating pluripotency and promoting the acquisition of an 8C‐like state, providing a useful in vitro system for exploring the regulatory mechanisms of stem cell plasticity.

## Graphical abstract

BCL2 stabilizes human naïve pluripotency associated with H3K27ac modulation and induces 8CLCs in human naïve ESCs.

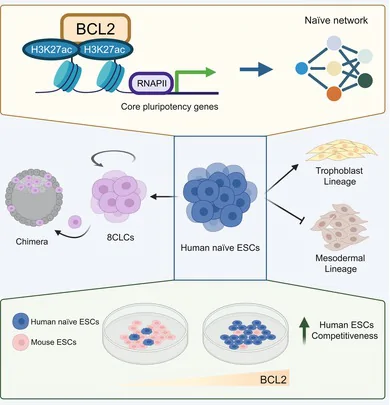

## 1 Introduction

Embryonic development begins with a totipotent zygote, which possesses the ability to give rise to all tissues and organs of the organism, including the placenta [1]. In mice, totipotency is restricted to the zygote and the two‐cell (2C) stage embryo [2, 3]. In humans, it is generally accepted that totipotency extends to the eight‐cell (8C) stage embryo [4, 5]. During mammalian embryonic development, the precise transition from the maternal genomes to the activation of the zygotic genome following fertilization plays a critical role in subsequent embryogenesis [6, 7]. Zygotic genome activation (ZGA) occurs at the two‐cell stage in mice and the eight‐cell stage in human embryos [6, 8]. As a key event in early development, understanding the molecular mechanisms regulating ZGA is important for human reproductive health. However, due to limitations in embryo availability and ethical considerations, it remains challenging to obtain a sufficient number of human embryos for research purposes. Therefore, embryonic stem cells serve as an ideal model system for studying early embryonic development in vitro.

In recent years, remarkable achievements have been made in human embryonic stem cell (hESCs) research, including the development of various culture systems designed to maintain naïve pluripotency [9, 10, 11], totipotent‐like properties [12, 13, 14], and intermediate states [15, 16, 17, 18], alongside the establishment of multiple types of human embryonic stem cell lines [19, 20]. Naïve pluripotent stem cells correspond to cells of the pre‐implantation epiblast, whereas primed stem cells represent post‐implantation‐stage cells [21, 22]. Moreover, in both mouse and human naïve embryonic stem cells, a rare subpopulation of cells termed 2C‐like cells (2CLCs) and 8C‐like cells (8CLCs) has been identified [4, 5, 23, 24, 25, 26]. These cells exhibit a transcriptional and functional profile closely resembling that of the totipotent 2‐cell embryo in mice and the 8‐cell embryo in humans, mirroring key aspects of early embryonic potency in vivo. The generation of human 8CLCs has been achieved through several established methods [5, 27]. These include the identification and derivation of 8CLCs from naïve human embryonic stem cells through methods such as chemical induction or transgenic approaches [5, 27, 28]. These 8CLCs possess a transcriptome closely resembling that of human 8‐cell stage embryos and consistently express key genes linked to zygotic genome activation [4].

Meanwhile, an alternative approach has been reported, in which a transgene‐free chemical induction method was used to generate human 8CLCs from both primed and naïve state ESCs [5]. In addition, splicing inhibition has been shown to transiently reprogram human pluripotent stem cells into zygotic genome activation‐like cells (ZLCs), which further transition into stable totipotent blastomere‐like cells (TBLCs) upon long‐term passaging [14]. In contrast to 8CLCs, both ZLCs and TBLCs exhibit broad silencing of pluripotency‐associated genes [14]. Notably, TBLCs are capable of recapitulating key events of human pre‐implantation development during in vitro differentiation and demonstrate dual potential to contribute to both embryonic and extraembryonic lineages [14]. However, the regulatory mechanisms underlying human zygotic genome activation and the induction and maintenance of 8C‐like cells in human embryonic stem cells remain poorly understood.

We have previously demonstrated that BCL2, either alone or in combination with NELFA, induces a 2C‐like state in mouse ESCs [29]. Human ESCs overexpressing the anti‐apoptotic genes *BCL2* or *BCL2L1* have been shown to contribute to interspecific human–mouse chimeras following injection into mouse embryos [30]. Therefore, we asked whether the function of BCL2 is conserved in human 8C‐like cells induction. In this study, we demonstrate that overexpression of *BCL2* enhances the pluripotency of human naïve ESCs. Notably, *BCL2* overexpression facilitated the long‐term and stable maintenance of human 8CLCs. Our study thus provides critical insights into the regulation of human zygotic genome activation and establishes a functional framework for understanding totipotency and lineage specification in early human development.

## 2 Material and Methods

### 2.1 Human Primed ESCs Culture

Human primed ESCs, H9, were routinely cultured in mTeSR1 medium (Stemcell Technologies, 85850) on plates coated with Matrigel (Corning, 354230) according to the manufacturer's protocol. Generally, ESCs were passaged every 3–4 days and medium was refreshed every day. For passaging, cells were washed with DPBS (Thermo Fisher, 14200075) once and treated with TrypLE Express (Gibco, 12563029) for 5 min. Then, TrypLE was removed, and cells were passaged as small clumps. The cells were cultured in an incubator at 37°C and 5% CO_2_. All cell lines were confirmed as mycoplasma negative.

### 2.2 Generation of *BCL2* Overexpression Human Primed ESCs

*BCL2* overexpression human primed ESCs (*BCL2*_OE ESCs) or human chemically induced pluripotent stem cell (CiPSCs) lines were generated by Lipofectamine Stem Transfection Reagent (Thermo Fisher, STEM00008) of ESCs or CiPSCs with PB‐CAG‐BCL2‐mCherry (2 μg) and PB‐EF1α‐transposase (2 μg). After transfection, cells were treated with 1 μg/mL puromycin (MedChemExpress, HY‐B1743) for 24–48 h for selection. The remaining cells were split into 96‐wells using mTeSR 1 medium (Stemcell Technologies, 85850) with 10 μM Y‐27632 (MedChemExpress, HY‐10071). Y‐27632 was withdrawn 24 h after splitting, and the medium was refreshed every day. Culture wells containing a single colony were selected and expanded, and confirmed by Sanger sequencing.

### 2.3 Human ESCs Primed to Naïve Conversion

To generate 4CL naïve ESCs or CiPSCs [5], wild‐type and *BCL2*_OE cells were washed with DPBS once, dissociated into single cells with TrypLE Express (Gibco, 12563029) and plated at a density of 1000–1500 cells cm^2^ on Matrigel‐coated plates in mTeSR 1 medium supplemented with 10 μM Y‐27632. Twenty‐four hours later, the medium was switched to 4CL medium composed of a 1:1 mix of Neurobasal medium (Gibco, 21103049) and Advanced DMEM/F12 (Gibco, 12634010) supplemented with 1× N2 supplement (Gibco, 17502048), 1× B27 supplement (Gibco, 17504044), 1% Glutamax (Gibco, 35050061), 1% MEM NEAA (Gibco, 11140050), 100 U penicillin/streptomycin (Gibco, 15140122), 1 mM sodium pyruvate (Gibco, 11360070), 0.1 mM β‐mercaptoethanol (Gibco, 21985023), 10 nM DZNep (Selleck, S7120), 5 nM TSA (Sigma, T8552), 1 μM PD0325901 (Miltenyi biotec, 130‐103‐923), 5 μM IWR‐1 (Selleckchem, S7086), 20 ng/mL Human LIF (Peprotech, 300‐05), 20 ng/mL ACTIVIN A (Peprotech, 338‐AC), 50 μg/mL L‐ascorbic acid (Sigma, 49752) and 0.2% (v/v) Matrigel. The 4CL culture medium was replaced daily, and the cells were passaged in the form of single cells every 3–4 days. The cells were cultured in an incubator at 37°C and 5% CO_2_.

### 2.4 Generation of Human 8CLCs From Naïve ESCs

To generate human 8CLCs in stepwise e4CL [5], wild‐type and *BCL2*_OE ESCs or CiPSCs were dissociated into single cells and plated at 2000–3000 cells cm^2^ on Matrigel coated plates in 4CL medium and replaced it with e4CL medium 24 h later. The composition of e4CL is the same as 4CL except for higher concentrations of DZNep (50 nM) (Selleck, S7120) and TSA (20 nM) (Sigma, T8552). e4CL medium was refreshed every day until day 6. The cells were cultured in an incubator at 37°C and 5% CO_2_.

### 2.5 Human ESCs In Vitro Differentiation

Pre‐mesendoderm (Pre‐ME), ME and definitive endoderm (DE) were induced from wild‐type and *BCL2*_OE ESCs using the aRB27 basal medium, which was composed of Advanced RPMI 1640 Medium (Gibco, 12633012) supplemented with 1% B27 supplement (Gibco, 17504044), 1% Glutamax (Gibco, 35050061), 1% MEM NEAA (Gibco, 11140050), 100 U penicillin/streptomycin (Gibco, 15140122). To induce ME, trypsinized ESCs were seeded on a vitronectin‐coated dish at 200,000 cells per well in a 12‐well plate and cultured in ME induction medium for 12 h (PreME) and 24 h (ME). ME induction medium contained aRB27 medium supplemented with 100 ng/mL ACTIVIN A (Peprotech, 338‐AC), 3 μM CHIR99021 (Miltenyi Biotec, 130‐103‐926) and 10 μM Y‐27632 (MedChemExpress, HY‐10071). To induce DE from ME, ME induction medium was replaced with a DE induction medium after washing with PBS once, and cells were cultured for a further 2 days. DE induction medium was composed of aRB27 medium supplemented with 100 ng/mL ACTIVIN A (Peprotech, 338‐AC) and 0.5 μM BMP inhibitor LDN193189 (Sigma, SML0559).

To induce hPGCLCs, PreME cells were trypsinized and plated into Corning Costar Ultra‐Low attachment 96‐well plate at 4000 cells per well in hPGCLC induction medium composed of aRB27 medium supplemented with 500 ng/mL BMP4 (R&D Systems, 314‐BP), 10 ng/mL Human LIF (Peprotech, 300‐05), 100 ng/mL SCF (R&D Systems, 11010‐SC), 50 ng/mL EGF (R&D Systems, 236‐EG), 10 μM ROCKi (MedChemExpress, HY‐10583), and 0.25% (v/v) poly‐vinyl alcohol. Cells were cultured as floating aggregates for 4 days.

For TS‐like cell induction, we used N2B27 basic medium supplemented with 1 μM PD0325901 (Miltenyi biotec, 130‐103‐923), 2 μM XAV939 (Sigma, X3004), 2 μM Gö6983 (Selleck, S2911), 10 ng/mL Human LIF (Peprotech, 300‐05), 5 ng/mL ACTIVIN A (Peprotech, 338‐AC), 10 ng/mL BMP4 (R&D Systems, 314‐BP). The cells were cultured on matrigel‐coated plate in an incubator at 37°C and 5% CO_2_.

### 2.6 Mouse and Human Embryonic Stem Cells Co‐Cultures

Mouse and human embryonic stem cells were seeded onto FN‐coated plates and either cultured mixed at 1:1 ratio for co‐cultures. For mouse naïve ESCs culture condition co‐culture experiments, human and mouse ESCs were co‐cultured in 2i/LIF medium. 2i/LIF medium contains DMEM/F12 and Neurobasal medium mixed at 1:1 ratio, 1× N2 supplement (Gibco, 17502048), 1× B27 supplement (Gibco, 17504044), 1% BSA, 15% KSR (Gibco, 2861518), 1% Glutamax (Gibco, 35050061), 1% MEM NEAA (Gibco, 11140050), 100 U penicillin/streptomycin (Gibco, 15140122), 0.1 mM β‐mercaptoethanol (Gibco, 21985023), 1000 IU/mL LIF (Millipore, ESG1107), 1 μM PD0325901 (Miltenyi Biotec, 130‐103‐923), and 3 μM CHIR99021 (Miltenyi Biotec, 130‐103‐926). For mouse EpiSCs culture condition co‐culture experiments, human ESCs and mouse EpiSCs were cultured in N2B27 based AF medium with 20 ng/mL ACTIVIN A (Peprotech, 338‐AC) and 12 ng/mL bFGF (MedChemExpress, HY‐P5321). The percentages of each cell line were determined using the Flow Cytometer (Cytoflex LX).

### 2.7 Flow Cytometry

For human PGCLCs, day 4 embryoid bodies (EBs) were trypsinized with 0.25% trypsin at 37°C for 15 min. Cell suspension was diluted with FACS medium (PBS with 3% fetal bovine serum and 5 mM EDTA) and centrifuged at 500 g for 5 min. The cell pellet was suspended with FACS medium and incubated with antibodies for 20 min at room temperature in the dark. Human PGCLCs expressing the TNAP (BD, 742712) and PDPN (BD, 749722) proteins were harvested and sorted using the BD FACSAria Sorp cell sorter. PGCLCs lacking TNAP or PDPN were used as negative controls, and FACS plots were generated by FlowJo (10.7.1).

### 2.8 CRISPRa

We designed a piggyBAC‐based CRISPRa (puromycin‐resistant cassette) doxycycline (Dox)‐inducible SunTag system that consists of a dCas9 fused to five GCN4 peptides separated by optimized 22‐amino‐acid linkers and an scFV‐sfGFP‐VP64 transactivator fusion peptide that can be recruited to the dCas9 through the scFV‐GCN4 domains^2^. The system is completed with piggyBAC gRNA plasmid, which entails a sgRNA cassette driven by a U6 promoter and a Tet‐On 3G‐IRES2‐neomycin resistance cassette driven by an EF1α promoter. To improve sgRNA expression level and stability, we adopted an optimized scaffold sequence with an A‐U basepair flip in the sgRNA stem‐loop and an extended hairpin structure as described previously [31]. Three sgRNAs targeting the *BCL2* promoters and three non‐targeting sgRNA controls were designed using CRISPOR Cas9 gRNA design tool (https://crispor.gi.ucsc.edu/crispor.py). The piggyBAC‐based CRISPRa (puromycin‐resistant cassette) and sgRNA plasmids (neomycin‐resistant cassette), together with a plasmid encoding a hyperactive piggyBAC transposase, were co‐transfected into human primed ESCs using the Lonza 4D‐Nucleofector transfection device. Stable cell lines with integration of the CRISPRa and sgRNA transgenes were generated after puromycin (MedChemExpress, HY‐B1743) and neomycin (Gibco, 10131035) selection for 7–10 days. To activate the promoters, cells were treated with 1 μg/mL Dox in mTeSR1 medium for 3 days and performed RT‐qPCR and western blot analysis. All sgRNA sequences are listed in Table S13.

### 2.9 RNA Sequencing (RNA‐Seq) and Data Analysis

Total RNA (mTeSR‐WT, p38; mTeSR‐*BCL2*_OE, p15; 4CL‐WT, p15; 4CL‐*BCL2*_OE, p15; e4CL‐WT, day 6; e4CL‐*BCL2*_OE, day 6; e4CL‐WT, day 18; e4CL‐*BCL2*_OE, day 18) was extracted with the RNeasy Plus Mini Kit (Qiagen, 74104) according to the manufacturer's protocol. mRNA was purified from total RNA using poly‐T oligo‐attached magnetic beads. Fragmentation was performed using divalent cations under elevated temperature in 5× NEBNext first strand synthesis reaction buffer. First‐strand cDNA was synthesized using random hexamer primers and M‐MuLV reverse transcriptase. Second‐strand cDNA synthesis was performed using DNA Polymerase I and RNase H. Overhangs were converted to blunt ends via exonuclease/polymerase activity. The library fragments were purified with the AMPure XP system (Beckman Coulter). Sample clustering was performed on a cBot Cluster Generation System using the TruSeq PE Cluster Kit v3‐cBot‐HS (Illumina) according to the manufacturer's instructions. Subsequently, the library preparations were sequenced on an Illumina Novaseq platform and paired‐end reads (150 bp) were generated.

After sequencing, the paired‐end clean reads were aligned to the reference genome (UCSC GRCh38/hg38) using Hisat2 v2.0.5. Feature Counts v1.5.0‐p3 was used to quantify the number of reads mapped to each gene. Then, the FPKM was calculated for each gene based on the gene length and read count. Differential expression analysis was performed using the DESeq2 R package (1.16.1) with fold change ≥ 2 and *p*‐value < 0.05. UHC analysis was performed by the R hclust function. Heatmaps of select genes were performed using the R *heatmap.2* function. Principal component analysis was performed with the R *prcomp* function. Gene ontology (GO) enrichment analysis of differentially expressed genes (DEGs) was implemented using the clusterProfiler R package, with correction for gene length bias. GO terms with a corrected *p*‐value < 0.05 were considered significantly enriched by differentially expressed genes.

### 2.10 CUT&Tag‐Seq Sample Preparation

Cultured human ESCs (4CL‐WT, p28; 4CL‐*BCL2*_OE, p30; e4CL‐*BCL2*_OE, p12) were harvested and centrifuged at 500 × g for 5 min at 4°C. Cell viability and concentration were determined using the LUNA‐FL automated cell counter. Subsequently, 500,000 live cells were pelleted by centrifugation at 600 × g for 5 min. The pellet was resuspended in 500 μL of wash buffer to form a single‐cell suspension for the subsequent experiment.

### 2.11 CUT&Tag‐Seq Library and Sequencing

CUT&Tag library was performed as described previously [32]. Briefly, the cells are bound to Concanavalin A‐coated magnetic beads, and the cell membrane is permeabilized by Digitonin. The enzyme pA‐Tn5 Transposase precisely binds the DNA sequence near the target protein under the antibody guidance and results in factor‐targeted tagmentation. DNA sequence is tagmented, with adapters added at the same time at both ends, which can be enriched by PCR to form the sequencing‐ready libraries (Vazyme, TD904). After the PCR reaction, libraries were purified with the AMPure beads and library quality was assessed on the Agilent Bioanalyzer 2100 system. The clustering of the index‐coded samples was performed on a cBot Cluster Generation System using TruSeq PE Cluster Kit v3‐cBot‐HS (Illumina) according to the manufacturer's instructions. The library preparations were sequenced on the Illumina Novaseq platform at Novogene Co. Ltd. (Beijing, China), and 150 bp paired‐end reads were generated.

### 2.12 CUT&Tag‐Seq Data Processing

Raw data (raw reads) of fastq format were firstly processed using fastp (v0.20.0) [33]. In this step, clean data (clean reads) were obtained by removing reads containing adapter, reads containing ploy‐N, and low‐quality reads from raw data. At the same time, Q20, Q30, and GC content of the clean data were calculated. All the downstream analyses were based on the clean data. Reference genome (UCSC GRCh38/hg38) and gene annotation files were downloaded from genome website directly. Index of the reference genome was built using BWA (v0.7.12), and clean reads were aligned to the reference genome using BWA mem ‐k 32 ‐T 30 ‐t 4 ‐M. These reads were then filtered for high quality (MAPQ ≥ 13); only uniquely mapped reads were used for further analysis.

### 2.13 CUT&Tag‐Seq Reproducible Peak Calling

All peak calling was performed with MACS2 (v2.1.0) using ‘macs2‐q 0.05‐f AUTO‐call summits‐nomodel‐shift‐100‐extsize 200‐keep‐dup all’ [34]. By default, peaks with a *q*‐value threshold of 0.05 were used for all data sets.

### 2.14 CUT&Tag‐Seq Motif Analysis, Peak Annotation and Different Peak Analysis

Peaks were adjusted to the same size (500 bp) centered peak summits and motif discoveries of these loci sequences were performed using findMotifsGenome.pl. program in HOMER (v4.9.1) software with ‘‐len 8,10,12,14‐gc‐size given‐homer2‐dumpFasta’ [35]. The position of peak summit around transcript start sites of genes can predict the interaction sites between protein and gene. ChIPseeker was used to retrieve the nearest genes around the peak and annotate the genomic region of the peak [35]. Peak‐related genes can be confirmed by ChIPseeker, and then Gene Ontology (GO) enrichment analysis was performed to identify the functional enrichment results. GO enrichment analysis was implemented by the GOseq R package, in which gene length bias was corrected. GO terms with corrected *p*‐value less than 0.05 were considered significantly enriched by peak‐related genes [36]. Peaks of different groups were merged using ‘bedtools merge’ [37]. We calculated the mean RPM of each group in the merged peak. Only peaks with fold change of RPM more than 2 were considered as differential peaks. Genes associated with different peaks were identified using ChIPseeker.

### 2.15 Human‐Mouse Chimeric Experiments

Mice were kept in the animal house of Inner Mongolia University, China. Mouse handling and experiments were carried out with humane methods in compliance with the guidelines of the Institutional Animal Care and Use Committee, Inner Mongolia University (SYXK(MENG)2020‐0006). 8‐cell stage embryos were obtained by flushing the oviducts of female ICR mice. Corresponding cells with reporter expression were dissociated, and 10–15 WT or *BCL2*_OE ESCs cultured in e4CL were injected into each embryo. Following microinjection, 8‐cell stage embryos were cultured in G‐2 PLUS (Vitrolife, 10132) for another 48–60 h at 37°C with 5% CO_2_. The chimeric blastocysts were fixed for immunostaining. For chimerism assessment, we performed immunostaining for OCT4 (ICM) and GATA3 (TE), and statistically analyzed OCT4 positive embryos as well as OCT4/GATA3 double positive embryos.

### 2.16 RT‐qPCR

The total RNA of human ESCs was extracted with the RNeasy Plus Mini Kit (Qiagen, 74104). cDNA was obtained by Reverse Transcription System (Qiagen, 205311). Real‐time PCR reactions were set up in duplicate using KAPA SYBR FAST qPCR kit (KAPA Biosystems, KK4601) and repeated with at least three biological replicates with similar results. GAPDH expression level was used as an internal normalization control. All samples were conducted using a LightCycler 96 Instrument (Roche Molecular Systems). Relative expression levels were assessed by the 2^−ΔΔ*Ct*^ method. All primers used in this study are listed in Table S13.

### 2.17 Immunofluorescence Staining

For immunofluorescence staining, human ESCs were fixed with 4% PFA in PBS for 30 min at room temperature and blocked with PBS containing 1% BSA and 0.1% Triton X‐100 for 30 min. Primary antibodies diluted in blocking buffer were applied to cells and incubated for overnight at 4°C. The antibodies used were: 1:100 goat anti‐SOX2 (R&D, AF2018); 1:100 rabbit anti‐OCT4 (NOVUSBIO, NB100‐2379); 1:100 rabbit anti‐NANOG (Peprotech, 500‐P236); 1:500 rabbit anti‐ZSCAN4 (Abcam, ab106646); 1:200 rabbit anti‐TPRX1 (Proteintech, 25834‐1‐AP); 1:100 goat anti‐GATA3 (R&D, AF2605); 1:100 rabbit anti‐ELF5 (Abclonal, A7181); 1:200 mouse‐anti CDX2 (BioGenex, AM392); 1:200 mouse anti‐TFAP2C (Santa Cruz Biotechnology, sc‐12762); rabbit anti‐PRDM1 (Cell Signalling Technology, 9115); 1:100 goat anti‐SOX17 (R&D, AF1924); 1:200 rabbit anti‐BRACHYURY (T) (Abcam, ab209665); 1:200 sheep anti‐EOMES (R&D, AF6166); 1:100 goat anti‐FOXA2 (R&D, AF2400); 1:100 goat anti‐GATA6 (R&D, AF1700); 1:100 rabbit anti‐BCL2 (ABclonal, A21592); Next, the samples were washed three times with PBS, followed by incubation with fluorescence‐conjugated secondary antibodies (Thermo Fisher Scientific) for 1 h at room temperature. After washing, the slides were then mounted in Vectashield with DAPI (Vector Laboratories). Samples were imaged under Leica SP8 upright or inverted scanning confocal microscope or Olympus FV1000 confocal microscope.

### 2.18 Western Blot Analysis

Total protein was extracted using lysis buffer (Solarbio, R0030) containing 0.1 M PMSF (Solarbio, P0100) and 10 g/L phosphatase inhibitor (Thermo Scientific, A32957) on ice. After centrifugation at 13,000 × g for 10 min at 4°C, the protein concentration in the supernatant was determined using a Pierce BCA Protein Assay Kit (Thermo Scientific, 23227). Protein was separated by 10%–12% SDS‐PAGE, transferred to a nitrocellulose membrane (BIO‐RAD, 1620177), blocked with 5% skimmed milk (BD, 232100) for 1 h at room temperature, and then incubated first with primary antibody overnight at 4°C, and then with secondary antibody for 1 h at room temperature. The antibodies used were: 1:1000 rabbit anti‐OCT4 (NOVUSBIO, NB100‐2379); 1:1000 rabbit anti‐NANOG (Peprotech, 500‐P236); 1:1000 goat anti‐SOX2 (R&D, AF2018); 1:1000 rabbit anti‐ZSCAN4 (Abcam, ab106646); 1:1000 rabbit anti‐TPRX1 (Proteintech, 25834‐1‐AP); 1:500 rabbit anti‐DUX (HUABIO, ER2001‐60); 1:1000 rabbit anti‐NELFA (Bethyl Laboratories, A301‐910A); 1:1000 mouse anti‐p53 (BD Biosciences, 554293); 1:1000 rabbit anti‐BCL2 (ABclonal, A21592); 1:1000 rabbit anti‐H3K27ac (Active motif, 39133); 1:1000 rabbit anti‐H3K27me3 (Merck. 07‐449); 1:1000 rabbit anti‐H3 (Abcam, ab1791); 1:1000 goat anti‐DPPA5 (R&D, AF3125); 1:1000 rabbit anti‐EOMES (Santa Cruz, sc‐98555); 1:1000 goat‐anti HAND1 (R&D, AF3168); 1:5000 mouse anti‐β‐ACTIN (Elabscience, PKSH033261). The signals were visualized with Pierce ECL Western Blotting Substrate (Thermo Scientific, 32209) and detected with the ChemiDoc Touch Imaging System (Bio‐Rad). The uncropped original western blot images are provided in Table S14.

### 2.19 Statistical Analysis

All values are presented as mean ± SD. Statistical parameters including statistical analysis, statistical significance, and n value are reported in the Figure legends. Statistical analyses were performed using Prism Software (GraphPad Prism version 9). Differences between groups were evaluated by an unpaired two‐tailed Student's *t*‐test. A value of *p* value smaller than 0.05 was considered significant.

## 3 Results

### 3.1 BCL2 Stabilizes Naïve Pluripotency of Human ESCs Associated With H3K27ac

To explore the role of BCL2 in human primed ESCs, we generated stable *BCL2*‐overexpressing human primed ESCs (*BCL2*_OE ESCs) using a piggyBac transposon system under the control of a CAG promoter, with mCherry serving as a fluorescent reporter (Figure S1A). We found that ectopic expression of *BCL2* did not alter the morphological features of human primed ESCs (Figure S1B–D). Quantitative PCR (qPCR) analysis revealed that *BCL2* overexpression led to only a slight upregulation of pluripotency‐associated genes (including *OCT4*, *NANOG*, and *SOX2*) in *BCL2*_OE ESCs (Figure S1C), whereas the protein levels of these genes did not show significant changes tested by immunostaining (Figure S1E,F).

Next, to investigate whether *BCL2* overexpression alters the transcriptional profile of human primed ESCs, we performed RNA sequencing (RNA‐seq). In comparison to wild‐type primed ESCs (WT ESCs), *BCL2*_OE ESCs exhibited significant transcriptional changes, with 2252 genes upregulated and 2281 genes downregulated (log2FC > 1, padj < 0.05) (Figure S1G, and Table S1). We further focused on 587 previously identified primed pluripotency‐associated genes [5], and found that in *BCL2*_OE ESCs (compared with WT ESCs), approximately half of these genes trended toward upregulation and the other half toward downregulation (Figure S1H; Table S2). As a result, we observed no global difference in the expression profile of primed pluripotency‐associated genes between *BCL2*_OE ESCs and WT ESCs. Additionally, while no global difference in primed pluripotency‐related gene expression was detected overall, the expression of specific core pluripotency genes (*NANOG, POU5F1, KLF2, KLF4* and *DPPA3*) was slightly upregulated in *BCL2*_OE ESCs relative to WT ESCs, whereas that of another subset of pluripotency genes (*MYC, ESRRB, FGF4, TFCP2L1* and *TFAP2C*) was downregulated (Figure S1I). To further explore the functional implications of these transcriptional changes, we performed gene set enrichment analysis (GSEA), which showed that genes upregulated in *BCL2*_OE ESCs relative to WT ESCs were enriched in processes associated with the negative regulation of apoptotic signalling pathway and regulation of signal transduction by p53 (Figure S1J). Thus, *BCL2* overexpression exerts only a limited effect on maintaining pluripotency in human primed embryonic stem cells.

To clarify whether BCL2 has distinct functional roles in primed versus naïve human ESCs, we first reprogrammed *BCL2*‐overexpressing human primed ESCs to the naïve pluripotent state using the established 4CL medium (Figure 1A) [5]. We found that compared to WT ESCs, *BCL2*_OE ESCs exhibited a closer morphological resemblance to naïve‐like ‘dome‐shaped’ colonies (Figure 1B). However, morphological similarity alone is insufficient to confirm that the cells have acquired a naïve pluripotent identity. Consequently, we conducted gene expression profiling and observed that *BCL2*_OE ESCs exhibited significantly elevated expression of core pluripotency factors (*OCT4, NANOG, MYC*) and naïve pluripotency markers (*TFCP2L1, DNMT3L, KLF4*) compared with WT ESCs (Figure 1C,D). To further confirm these transcriptional findings, we performed western blot analysis to assess protein expression, which demonstrated notably higher levels of OCT4, NANOG, and SOX2 in *BCL2*_OE ESCs versus WT ESCs (Figure 1E; Figure S1K).

**FIGURE 1:**
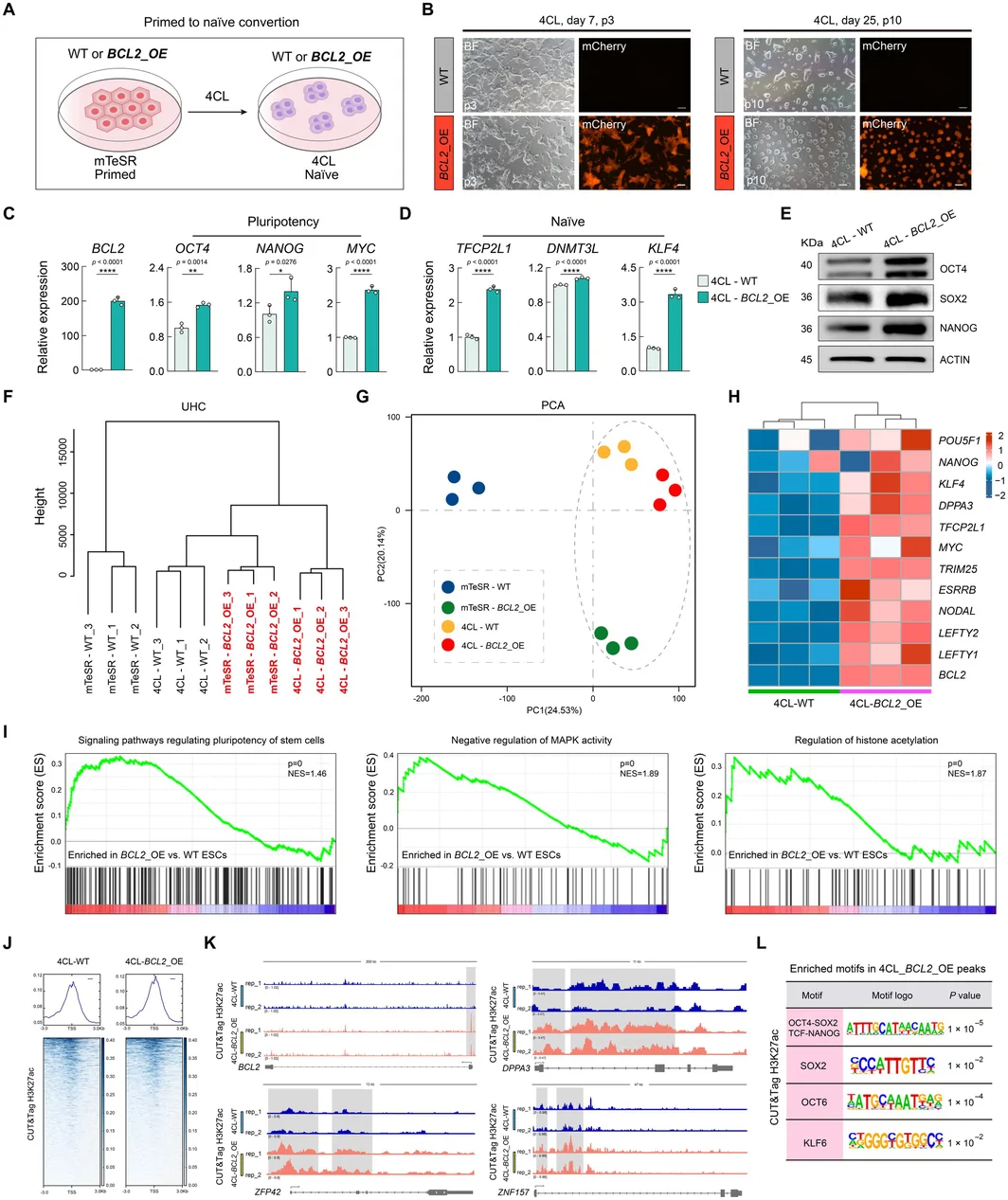
BCL2 stabilizes naïve pluripotency of human ESCs associated with H3K27ac. (A) Schematic of experimental design for converting human primed ESCs to naïve state using 4CL medium. (B) Representative morphology of WT and *BCL2*_OE ESCs in 4CL medium at day 7 (left) and day 25 (right). Scale bar, 100 μm. (C, D) qPCR analysis of core pluripotency genes (*OCT4*, *NANOG*, *MYC*) (C) and naïve‐state markers (*TFCP2L1*, *DNMT3L*, *KLF4*) (D) in WT and *BCL2*_OE ESCs cultured in 4CL medium at day 25 (passage 10, p10). Error bars represent means ± SD (*n* = 3 biological replicates). The *p*‐values were calculated using two‐tailed Student's *t*‐tests (C, D), *p < 0.05, **p < 0.01, ****p < 0.0001, ns: Not significant. (E) Western blot analysis of OCT4, NANOG, and SOX2 protein levels in WT and *BCL2*_OE ESCs cultured in 4CL medium at day 25 (passage 10, p10). (F, G) Unsupervised hierarchical clustering (F) and principal component analysis (G) of RNA‐seq data from WT and *BCL2*_OE ESCs under different culture conditions (mTeSR‐WT, p38; mTeSR‐*BCL2*_OE, p15; 4CL‐WT, p15; 4CL‐*BCL2*_OE, p15). (H) Heatmap showing expression levels of key naïve pluripotency genes in WT and *BCL2*_OE ESCs. (I) Gene set enrichment analysis (GSEA) of biological processes enriched in *BCL2*_OE versus WT ESCs, cultured in 4CL medium. (J) Genome‐wide correlation of H3K27ac profiles between WT and *BCL2*_OE ESCs in 4CL medium (4CL‐WT, p28; 4CL‐*BCL2*_OE, p30). (K) H3K27ac CUT&Tag tracks at promoter and gene body regions of core pluripotency genes *DPPA3*, *ZFP42*, and *ZNF157* in WT and *BCL2*_OE ESCs. (L) Motif enrichment analysis of transcription factor motifs in differential H3K27ac peaks from *BCL2*_OE ESCs.

To further characterize the features of naïve pluripotency, we performed RNA sequencing (RNA‐seq) on WT and *BCL2*‐overexpressing ESCs cultured in 4CL medium. Comparative analysis revealed that 219 genes were significantly upregulated and 248 genes were downregulated in *BCL2*_OE ESCs relative to WT ESCs (Figure S1L; Table S3). Unsupervised hierarchical clustering (UHC) and principal component analysis (PCA) revealed that *BCL2*_OE cells clustered closely with WT ESCs cultured in 4CL medium, suggesting that they share key naïve pluripotency features (Figure 1F,G; Table S4). Interestingly, *BCL2*_OE cells cultured in mTeSR also co‐clustered with their 4CL‐cultured counterparts, demonstrating the robust capacity of BCL2 to induce features of naïve pluripotency (Figure 1F,G). However, the global expression profile of 124 known naïve genes [5] in *BCL2*_OE cells showed no significant difference compared to WT ESCs, with a mixed pattern of up‐ and down‐regulation (Figure S1M; Table S5). Notably, a subset of key naïve pluripotency genes including *POU5F1, NANOG, KLF4, DPPA3*, and *TFCP2L1* are significantly upregulated in *BCL2*_OE ESCs compared to WT ESCs in 4CL condition (Figure 1H). Gene set enrichment analysis (GSEA) showed that genes upregulated by *BCL2*_OE were enriched in terms related to signalling pathways regulating pluripotency of stem cells, negative regulation of MAPK activity, and regulation of histone acetylation compared to WT ESCs in 4CL condition (Figure 1I).

To explore the regulatory mechanism of BCL2, we hypothesized that the induction of naïve pluripotency mediated by BCL2 is associated with epigenetic regulation. To this end, we employed CUT&Tag sequencing to profile the genome‐wide distribution of the active histone mark H3K27ac. Although the global H3K27ac modification patterns were largely comparable between *BCL2*_OE and WT ESCs (Figure 1J), our analyses revealed that *BCL2*‐OE cells exhibited enhanced H3K27ac enrichment at the promoter region of the *BCL2* gene and at the loci of core pluripotency genes (*DPPA3, ZFP42, ZNF157*) (Figure 1K). Notably, this enrichment was primarily localized to promoter regions (Figure 1K). When we conducted motif enrichment analysis targeting the differential peaks in *BCL2*_OE cells, we detected enrichment of motifs for a suite of core pluripotency factors including OCT4‐SOX2‐TCF‐NANOG, SOX2, OCT6, and KLF6 (Figure 1L). Thus, our results support a model in which *BCL2* overexpression maintains naïve pluripotency of human ESCs in association with H3K27ac enrichment.

To assess the feasibility of loss‐of‐function approaches, we first examined endogenous BCL2 expression in human ESCs. Western blot analysis showed that BCL2 protein was undetectable in control cells without *BCL2* overexpression (Figure S1D). This was further confirmed by RNA‐seq data, which revealed extremely low *BCL2* read counts (40–70) in control ESCs, whereas *BCL2*_OE cells exhibited dramatically higher levels (1300‐1600) (Figure S1N), as well as by immunostaining (Figure S1O). Consistently, inducible CRISPRi‐mediated knockdown failed to achieve further effective suppression of *BCL2* due to its already minimal basal expression (Figure S1P–R). These results indicate that endogenous BCL2 is nearly absent in human ESCs under our experimental conditions, supporting that the observed phenotypes upon *BCL2* overexpression are attributable to BCL2 itself.

### 3.2 BCL2 Induces 8C‐Like State in Human Naïve ESCs

To assess whether *BCL2* overexpression promotes 8CLC induction in human naïve ESCs, we adopted e4CL medium, which contains higher doses of trichostatin A (TSA) and 3‐deazaneplanocin A (DZNep) than the base 4CL medium, in accordance with previously established protocols [5] (Figure 2A,B). Immunostaining analysis showed that 6‐day e4CL induction had minimal impact on the expression of key pluripotency proteins (OCT4, SOX2, NANOG) in *BCL2*_OE and WT ESCs (Figure 2C; Figure S2A). However, *BCL2* overexpression resulted in a significant upregulation of key naïve pluripotency (*DPPA3, DPPA5*, and *KLF4*) and 8CLC‐associated genes (*TPRX1, TRIM43, LEUTX, ZSCAN4, RFPL4A*, and *H3.X/Y*) compared to WT ESCs in 6 days e4CL induction (Figure 2D; Figure S2B). Consistent with this transcriptional signature, the protein levels of key 8CLC markers were also significantly upregulated in *BCL2*_OE ESCs relative to WT ESCs. This upregulation was validated at the protein level using two complementary approaches, immunostaining (for TPRX1 and ZSCAN4) and western blotting after 6 days e4CL induction (for TPRX1, DUX, ZSCAN4, DPPA5, and NELFA) (Figure 2E–G; Figure S2C).

**FIGURE 2:**
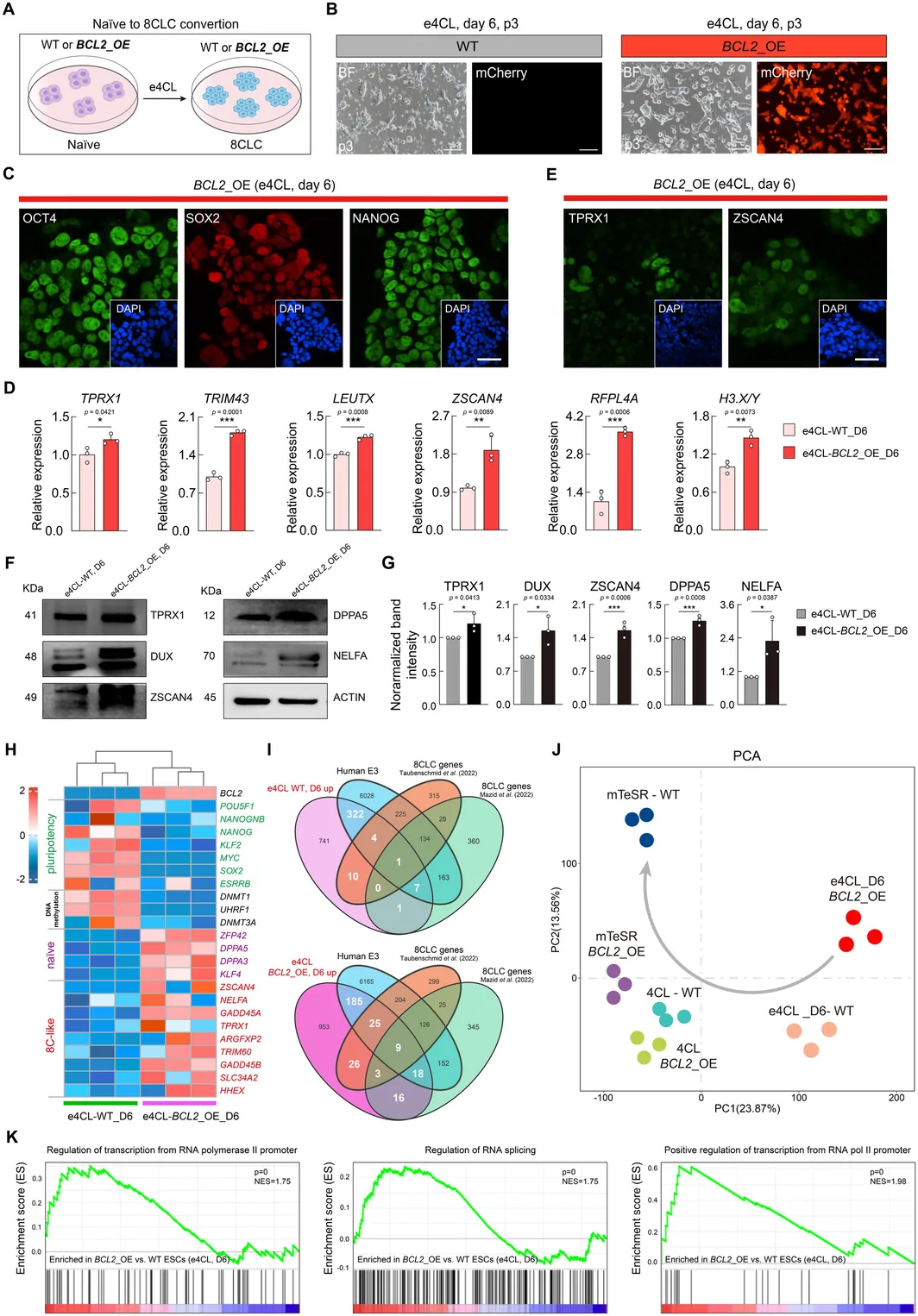
*BCL2* overexpression induces an 8C‐like state in human naïve ESCs. (A) Schematic diagram of the experimental setup for inducing 8C‐like cells (8CLCs) in human naïve ESCs using the e4CL induction protocol. (B) Representative images of WT and *BCL2*_OE human naïve ESCs after 6‐day e4CL induction. Scale bar, 100 μm. (C) Immunostaining of key pluripotency proteins (OCT4, SOX2, NANOG) in *BCL2*_OE ESCs following 6‐day e4CL induction. Scale bar, 50 μm. (D) Relative RNA expression levels of key 8CLC‐associated genes (*TPRX1, TRIM43, LEUTX, ZSCAN4, RFPL4A, H3.X/Y*) in WT and *BCL2*_OE ESCs after 6‐day e4CL induction. (E) Immunostaining of TPRX1 and ZSCAN4 in *BCL2*_OE ESCs after 6‐day e4CL induction. Scale bar, 50 μm. (F) Western blot analysis of TPRX1, DUX, ZSCAN4, DPPA5, and NELFA protein expression after 6 days e4CL induction. (G) Quantification of western blot results from three independent experiments of (F). (H) Heatmap showing selected significantly upregulated 8C‐like, naïve, DNA methylation, and pluripotency‐related genes in WT and *BCL2*_OE ESCs after 6‐day e4CL induction. (I) Comparative overlap analysis was performed between upregulated genes in *BCL2*_OE versus WT ESCs (cultured in e4CL, day 6) and published gene sets from human day 3 embryos and 8C‐like cells. (J) Principal component analysis (PCA) of transcriptomes from *BCL2*_OE and WT ESCs after 6‐day e4CL induction, showing transcriptional resemblance toward primed state. (K) Gene Set Enrichment Analysis (GSEA) showing significant enrichment of zygotic genome activation‐related terms in *BCL2*‐upregulated genes compared with WT ESCs after 6‐day e4CL induction. Error bars represent means ± SD (*n* = 3 biological replicates). The *p*‐values were calculated using two‐tailed Student's *t*‐tests (D, G), *p < 0.05, **p < 0.01, ***p < 0.001, ns: Not significant.

To delineate the molecular dynamics underlying e4CL‐induced 8C‐like state, we performed RNA‐seq on *BCL2*_OE and WT ESCs on day 6 e4CL induction. A comparative analysis identified 1386 upregulated and 1145 downregulated genes in *BCL2*_OE ESCs versus WT ESCs (Figure S2D; Table S6). *BCL2* overexpression significantly upregulated genes associated with totipotency, including core totipotent‐related genes *ZSCAN4, TPRX1, SLC34A2* and *GADD45A* (Figure 2H). Conversely, a modest downregulation was observed for pluripotency related genes such as *POU5F1, NANOG, KLF2* and *SOX2*, although some of these were not included in the list of significantly differentially expressed genes (DESeq2 padj ≤ 0.05, |log2FC| ≥ 1.0) (Figure 2H). A similar decreasing trend was also noted for DNA methylation‐related genes, including *DNMT1, UHRF1* and *DNMT3A* (Figure 2H). Meanwhile, a set of core naïve pluripotency genes, including *ZFP42*, *DPPA5*, *DPPA3* and *KLF4*, displayed a tendency to be upregulated (Figure 2H).

We next performed a comparative overlap analysis between the upregulated genes in *BCL2*_OE versus WT ESCs and published gene sets derived from human day 3 embryos and 8C‐like cells. The results showed that genes upregulated in *BCL2*_OE ESCs shared significantly more overlaps with published human day 3 embryo (E3) [4] and 8C‐like gene sets [4, 5] than those upregulated in WT ESCs when e4CL induction (Figure 2I; Table S7). The principal component analysis (PCA) results are also consistent with a transcriptional resemblance to the 8C‐like state in *BCL2*_OE ESCs, as compared to primed human ESCs (Figure 2J; Table S8). Importantly, gene set enrichment analysis (GSEA) revealed that *BCL2*‐upregulated genes (compared to WT ESCs) were enriched for terms such as regulation of transcription from RNA polymerase II promoter, regulation of RNA splicing, and positive regulation of transcription from RNA pol II promoter, all of which are biological processes linked to zygotic genome activation (Figure 2K). Collectively, our findings support a role for *BCL2* overexpression in the acquisition of an 8C‐like state in human naïve ESCs.

### 3.3 BCL2 Enables Self‐Renewal of 8C‐Like Cells Under Defined Conditions

Given that sustained activation and long‐term maintenance of a totipotent state remain challenging even for mouse 2C‐like cells under defined culture conditions, we sought to determine whether *BCL2* overexpression could facilitate the long‐term expansion and stabilization of the 8C‐like state under e4CL induction. Under prolonged e4CL culture, WT ESCs could not be passaged (p) beyond p6 (23 days), and ES‐like colonies were nearly undetectable (Figure 3A). By contrast, *BCL2*_OE ESCs exhibited robust self‐renewal, sustained ES‐like colonies for over p10, and could even be stably propagated for more than 15 passages (Figure 3A; Figure S3A). Furthermore, karyotype analysis of the long‐term cultured *BCL2*_OE ESCs at p10 passages confirmed a normal diploid karyotype (Figure S3B). In light of the observation that WT ESCs could not be cultured for longer than 23 days in e4CL medium, we exclusively selected day 18 cells for all subsequent comparative analyses. We further observed that, under prolonged e4CL culture conditions, *BCL2*_OE ESCs not only sustained high protein expression levels of OCT4, SOX2, and NANOG but also displayed significant transcriptional upregulation of most core 8C‐like genes, including *KLF17, TPRX1, TRIM43, DUX4, LEUTX, ZSCAN4, RFPL4A*, and *H3.X/Y* compared to WT ESCs (Figure 3B,C). We further validated the increased protein levels of a subset of these 8C‐like genes by western blotting (TPRX1, ZSCAN4, DUX, NELFA, TP53) and immunostaining (TPRX1, ZSCAN4), which was consistent with their transcriptional profiles (Figure 3D; Figure S3C,D). It should be noted that WT ESCs cultured in e4CL for 6 days were used for this comparison, as extended culture to 18 days did not yield sufficient cells for western blot analysis.

**FIGURE 3:**
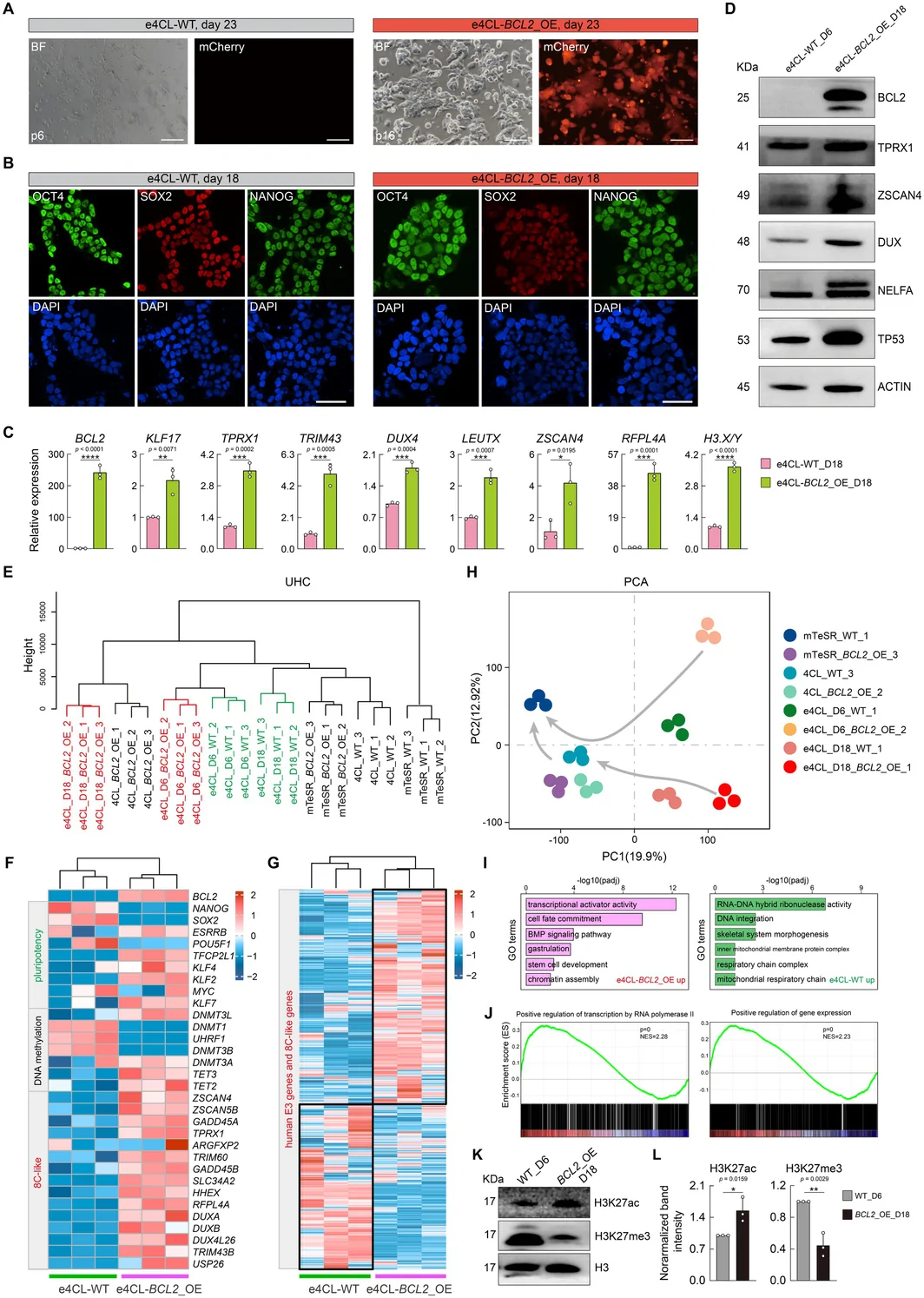
BCL2 maintains self‐renewal of 8C‐like cells under defined conditions. (A) Morphology of long‐term culture of WT and *BCL2*_OE ESCs under e4CL conditions. Scale bar, 100 μm. (B) Immunostaining of core pluripotency proteins (OCT4, SOX2, NANOG) in WT and *BCL2*_OE ESCs after prolonged e4CL culture (day 18). Scale bar, 50 μm. (C) Relative expression level of core 8C‐like genes (*KLF17,* *TPRX1, TRIM43, DUX4, LEUTX, ZSCAN4, RFPL4A, H3.X/Y*) in WT and *BCL2*_OE ESCs after 18‐day e4CL culture. (D) Western blot validation of selected 8C‐like markers (TPRX1, ZSCAN4, DUX, NELFA, TP53) in WT and *BCL2*_OE ESCs after 18‐day e4CL culture. (E) Unsupervised hierarchical clustering of transcriptomes from WT and *BCL2*_OE ESCs after e4CL culture with other different type cell lines. (F) Heatmap showing selected significantly upregulated 8C‐like, DNA methylation, pluripotency‐related genes in WT and *BCL2*_OE ESCs after 18‐day e4CL induction. (G) Heatmap showing published human day 3 (E3) and 8CLC‐specific genes in WT and *BCL2*_OE ESCs after 18‐day e4CL induction. (H) Principal component analysis showing transcriptional resemblance from 8C‐like state to primed state. (I) Gene ontology analysis of biological processes enriched in *BCL2*‐upregulated genes (left) and WT ESCs upregulated genes (right) after 18‐day e4CL induction. (J) Gene set enrichment analysis (GSEA) of biological processes enriched in *BCL2*_OE ESCs compared to WT ESCs after 18‐day e4CL induction. (K) Western blot analysis of H3K27ac and H3K27me3 levels in WT and *BCL2*_OE ESCs under e4CL conditions. (L) Quantification of western blot results from three independent experiments of (K). Error bars represent means ± SD (*n* = 3 biological replicates). The *p*‐values were calculated using two‐tailed Student's *t*‐tests (C, L), *p < 0.05, **p < 0.01, ***p < 0.001, ****p < 0.0001, ns: Not significant.

To further verify the stability of 8C‐like transcriptional profiles during long‐term propagation, we performed RNA‐seq analysis on WT and *BCL2*_OE ESCs after 18 days of e4CL culture. Unsupervised hierarchical clustering (UHC) analysis showed that *BCL2*_OE cells were clearly segregated from WT ESCs following 18 days of e4CL culture, demonstrating substantial transcriptional divergence between the two groups (Figure 3E; Table S9). A comparative analysis identified 1658 upregulated and 512 downregulated genes in *BCL2*_OE ESCs versus WT ESCs (Figure S3E; Table S10). As expected, our analysis of differentially expressed genes (DEGs) revealed that the majority of 8C‐like genes (*ZSCAN4, TPRX1, SLC34A2, RFPL4A, DUXA*, and others) exhibited persistently high expression levels in *BCL2*_OE ESCs relative to WT ESCs (Figure 3F). Furthermore, multiple core pluripotency genes (*TFCP2L1, KLF4, KLF2, KLF7*) displayed a notable upregulation tendency (Figure 3F). Heatmap‐assisted screening of our RNA‐seq data for published human day 3 (E3) [4] and 8CLC‐specific genes [4, 5] demonstrated that *BCL2*_OE ESCs harboured substantially more genes with marked upregulation relative to WT ESCs (Figure 3G; Table S11). Additionally, Venn overlap analysis revealed that genes upregulated in *BCL2*_OE ESCs (vs. WT ESCs) had significantly greater overlap with published human day 3 embryo and 8C‐like gene sets than the upregulated genes in WT ESCs (vs. *BCL2*_OE ESCs) (Figure S3F; Table S12).

Notably, PCA revealed that *BCL2*_OE ESCs exhibit a transcriptional resemblance to the 8C‐like state, whereas primed human ESCs are transcriptionally distinct (Figure 3H; Table S9). Gene ontology (GO) analysis revealed that *BCL2*‐upregulated genes were enriched for terms such as transcriptional activator activity, cell fate commitment, stem cell development, and chromatin assembly, all of which are biological processes linked to zygotic genome activation (Figure 3I). In contrast, genes highly expressed in WT ESCs showed no significant enrichment for these terms (Figure 3I). Gene set enrichment analysis (GSEA) reinforced this pattern by demonstrating significant enrichment in related processes, including the positive regulation of transcription by RNA polymerase II, positive regulation of gene expression, and chromosome (Figure 3J; Figure S3G).

Given the critical roles of epigenetic modifications in orchestrating embryonic development and cell fate specification, this study aimed to elucidate whether BCL2 is mechanistically linked to histone modifications during 8C‐like state activation. Western blot analysis revealed that *BCL2* overexpression significantly increased H3K27ac levels while concurrently decreasing H3K27me3 under e4CL conditions compared to WT ESCs (Figure 3K,L). These results strongly suggest that BCL2‐mediated activation of the 8C‐like state is linked to specific histone modifications. Taken together, our data solidly conclude that BCL2 is sufficient to sustain the continuous self‐renewal of the 8C‐like state during long‐term propagation under defined in vitro conditions.

### 3.4 Preservation of Key Pluripotency Genes Promotes Self‐Renewal and 8C‐Like State Activation in *BCL2* ‐Overexpressing ESCs

We next investigated the mechanistic basis for the long‐term self‐renewal of *BCL2*_OE ESCs in e4CL culture. We hypothesized that the activation of the 8C‐like state might coincide with the retention of core pluripotency features. Owing to the failure of WT ESCs to undergo long‐term passaging in e4CL medium, we performed H3K27ac CUT&Tag sequencing on p12 *BCL2*_OE ESCs maintained in e4CL medium, with *BCL2*_OE and WT ESCs in 4CL medium set as baseline controls. Consistent with our hypothesis, we detected pronounced H3K27ac enrichment peaks at both the promoters and intragenic regions of core pluripotency genes (*POU5F1, KLF4, BCL2L1, TFAP2C, DNMT3L, KLF5, BMP4*, and *JUP*) in *BCL2*_OE ESCs cultured in e4CL medium, indicative of an active histone modification state that is compatible with sustained self‐renewal (Figure 4A; Figure S4A). Motif enrichment analysis of the differential H3K27ac peaks in *BCL2*_OE cells in e4CL conditions also identified enrichment for motifs of core pluripotency transcription factors including OCT4‐SOX2‐TCF‐NANOG, OCT4, KLF4, TFAP2C, SOX2, and KLF5 (Figure 4B; Figure S4B). Gene set enrichment analysis (GSEA) of RNA‐seq data derived from *BCL2*_OE ESCs cultured in e4CL medium for 18 days revealed coordinated activation of pathways governing cell adhesion molecules, JAK–STAT signalling pathway, and WNT signalling pathway, relative to WT ESCs subjected to the same 18‐day e4CL culture condition (Figure S4C). These convergent results from transcriptomic and epigenomic analyses support that BCL2 establishes a stabilized 8C‐like state with retained pluripotency features.

**FIGURE 4:**
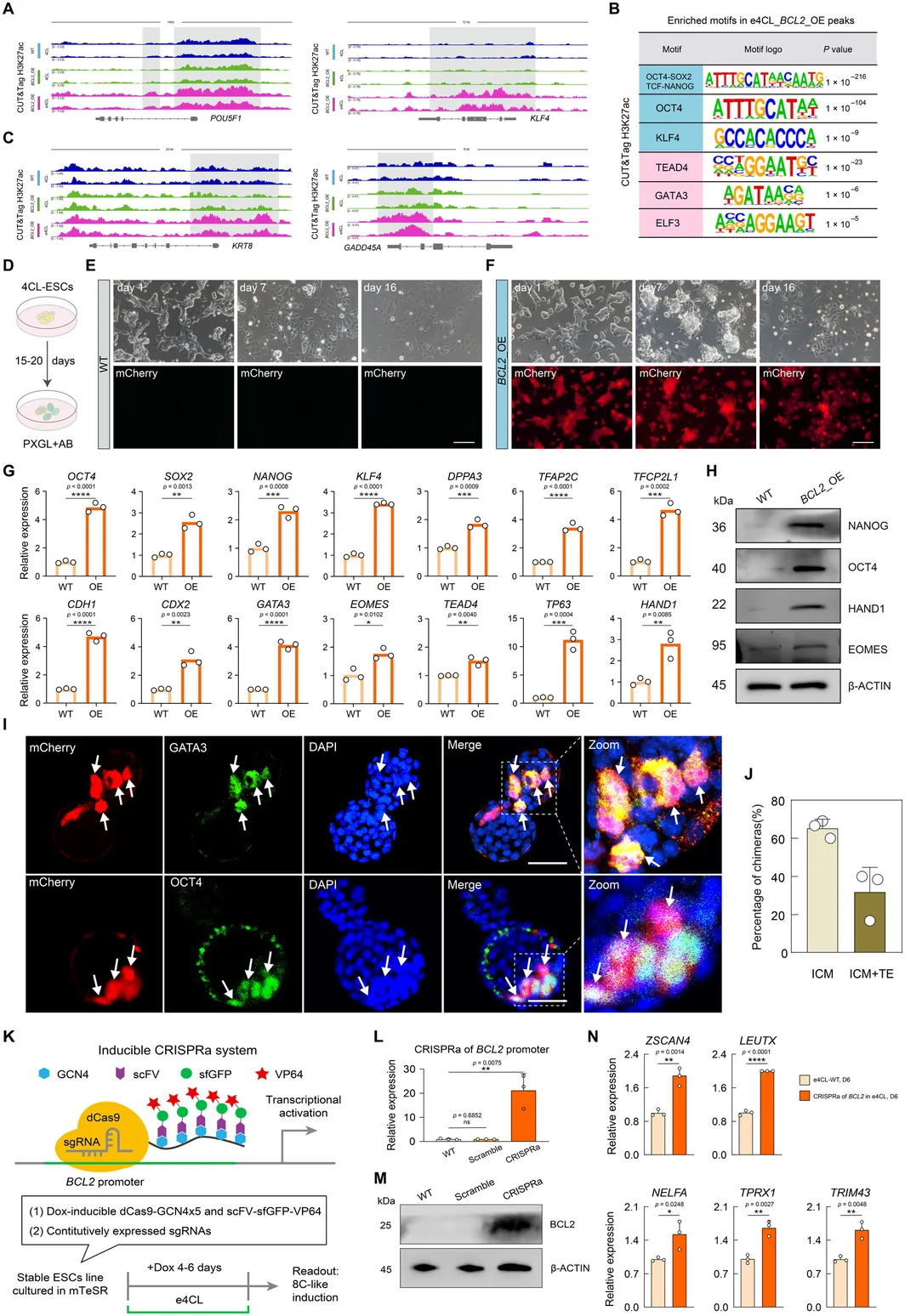
Dissecting the regulatory mechanism and functional properties of BCL2‐induced 8C‐like cells. (A) H3K27ac CUT&Tag peaks at promoter and gene body regions of core pluripotency genes (*POU5F1, KLF4*) in e4CL*‐BCL2*_OE ESCs (p12) cultured in e4CL medium compared to 4CL‐WT ESCs (p28) and 4CL‐*BCL2*_OE ESCs (p30). (B) Motif enrichment analysis of transcription factor motifs in differential H3K27ac peaks from e4CL*‐BCL2*_OE ESCs (p12) cultured in e4CL medium, showing enrichment for core pluripotency factors (top) and trophectoderm development factors (bottom). (C) H3K27ac CUT&Tag peaks at promoter and gene body regions of core trophectoderm‐like (TE‐like) genes (*KRT8, GADD45A*) in e4CL*‐BCL2*_OE ESCs (p12) cultured in e4CL medium compared to 4CL‐WT ESCs (p28) and 4CL‐*BCL2*_OE ESCs (p30). (D) Schematic diagram of the transition from human naïve ESCs to TE‐like cells in PXGL (with ACTIVIN A and BMP4) medium. (E) Bright field and fluorescence images showing the morphological changes from human naïve wild‐type ESCs to TE‐like cells at day 1, 7, and 16. Scale bar, 50 μm. (F) Bright field and fluorescence images showing the morphological changes from human naïve *BCL2*_OE ESCs to TE‐like cells at day 1, 7, and 16. Scale bar, 50 μm. (G) Relative expression levels of pluripotency (*OCT4*, *SOX2*, *NANOG*, *KLF4, DPPA3, TFAP2C, TFCP2L1*) and TE marker genes (*CDH1*, *CDX2, GATA3*, *EOMES, TEAD4, TP63, HAND1*) in human naïve wild‐type ESCs and *BCL2*_OE ESC‐derived TE‐like cells after 7 days induction, as quantified by RT‐qPCR. (H) Protein levels of pluripotency (NANOG, OCT4) and TE markers (HAND1, EOMES) detected by western blot in human naïve wild‐type ESCs and *BCL2*_OE ESC‐derived TE‐like cells after 7 days induction. (I) Representative confocal images of chimeric embryos showing mCherry‐labelled e4CL‐*BCL2*_OE ESCs contributing to both inner cell mass (ICM, OCT4+) and trophectoderm (TE, GATA3+). Scale bar, 50 μm. (J) Quantification of e4CL‐*BCL2*_OE ESCs contribution to ICM and TE lineages in chimeric embryos (*n* = 35). (K) Schematic of doxycycline (Dox)‐inducible CRISPRa system for endogenous *BCL2* activation. (L) Endogenous *BCL2* activation measured by qPCR following 3 days Dox induction. (M) Endogenous *BCL2* activation measured by western blotting. (N) Expression levels of 8CLC‐specific genes (*ZSCAN4, LEUTX, NELFA, TPRX1, TRIM43*) following endogenous *BCL2* activation in e4CL medium when 6 days Dox induction. Error bars represent means ± SD (*n* = 3 biological replicates). The *p*‐values were calculated using two‐tailed Student's *t*‐tests (G, L, N), *p < 0.05, **p < 0.01, ***p < 0.001, ****p < 0.0001, ns: Not significant.

Intriguingly, further analysis of differential H3K27ac peaks in *BCL2*_OE ESCs uncovered a subset of trophectoderm‐associated genes (*KRT8, KRT18, GADD45A*, and *RARG*), characterized by pronounced signals at promoter and gene body loci, suggesting potential lineage‐biased gene expression features (Figure 4C; Figure S4D). Motif enrichment analysis of the differential peaks in *BCL2*_OE cells also identified significant enrichment for motifs of trophectoderm development‐related factors including TEAD4, GATA3, ELF3, EOMES, EGR1, and HNF4A (Figure 4B; Figure S4E). The active histone modification state at trophectoderm‐associated genes was further supported by transcriptional profiling, which demonstrated their significant upregulation, particularly in *BCL2*_OE ESCs under e4CL conditions, relative to WT ESCs subjected to the same 18‐day e4CL cultures (Figure S4F). We therefore hypothesized that these properties of BCL2 may contribute to the conversion of human ESCs into trophoblast stem cell‐like cells (TS‐like cells). To assess the potential of BCL2 in trophoblast conversion, we differentiated 4CL cultured *BCL2*_OE and WT ESCs using a PXGL‐based medium supplemented with ACTIVIN A and BMP4 (Figure 4D). We found that TS‐like colony emergence was delayed in *BCL2*_OE ESCs compared to WT ESCs (Figure 4E,F). However, *BCL2*_OE ESCs exhibited significantly higher expression levels of both trophectoderm‐related and pluripotency‐associated genes at day seven TS‐like cells induction (Figure 4G,H; Figure S4G). These results suggest that BCL2 expands the differentiation potential of human naïve ESCs.

Next, we evaluated the in vivo developmental potential of e4CL cultured *BCL2*_OE ESCs by injecting them into 8‐cell stage embryos. Following 48‐h culture and immunostaining analysis, we observed that *BCL2* overexpressing cells contributed extensively to both the inner cell mass (65.7%, 23/35) and the trophectoderm (31.4%, 11/35) in chimeric embryos (Figure 4I,J). In contrast, WT ESCs e4CL cultured showed minimal contribution to the trophectoderm (3.3%, 1/30) (Figure S4H). These results demonstrate that BCL2 confers an expanded differentiation potential, enabling contribution to both embryonic and extra‐embryonic lineages under defined culture conditions. To complement the exogenous overexpression studies, we generated stable human naïve ESCs with Dox‐inducible CRISPR activation (CRISPRa) systems [31] targeting the endogenous *BCL2* locus (Figure 4K–M). Activation of endogenous *BCL2* in e4CL medium similarly induced the expression of 8CLC‐specific genes, with *ZSCAN4, LEUTX, NELFA, TPRX1*, and *TRIM43* being significantly upregulated compared to WT ESCs in day 6 e4CL cultures (Figure 4N). Our findings support a model where *BCL2* overexpression sustains human 8C‐like cells by preserving the expression of core pluripotency genes, thereby enabling their self‐renewal and lineage‐biased differentiation capacity.

### 3.5 Ectopic *BCL2* Expression Suppresses the Mesodermal Lineage Commitment of Human ESCs

Human embryonic stem cells possess the capacity to differentiate into mesendoderm (ME) through canonical WNT and ACTIVIN/NODAL signalling pathways [38, 39]. Around 12 h after induction, pre‐mesendoderm (PreME) cells transiently acquire competence for germ cell fate and can differentiate into human primordial germ cell‐like cells (PGCLCs) in response to BMP4 [40, 41]. When allowed to progress, PreME cells develop into mesendoderm (ME) by 12 h. Concurrently, they lose competence for the germ cell lineage and gain competence for mesoderm and definitive endoderm (DE) fates [42]. Next, we investigated whether *BCL2* overexpression influences the differentiation capacity of human primed ESCs. To this end, we assessed the PGCLC differentiation potential of *BCL2*_OE ESCs (Figure 5A). As reported previously, we assessed the potential of these cells to specify into PGCLCs [40]. While *BCL2* overexpression did not impair the ability of ESCs to form embryoid bodies (Figure 5B; Figure S5A), further gene expression analysis within the embryoid bodies revealed a distinct molecular profile. The expression of pluripotency genes *OCT4* and *NANOG* is significantly downregulated, whereas *SOX2* expression was elevated in *BCL2*_OE ESC‐derived PGCLCs compared with WT ESCs (Figure 5C). Concurrently, the expression levels of key primordial germ cell‐related genes including *SOX17, TFAP2C, NANOS3* and *PRDM1* were markedly reduced in *BCL2*_OE ESC‐derived PGCLCs compared with WT ESCs (Figure 5C). In line with the gene expression data, immunostaining further demonstrated a significant reduction of OCT4, PRDM1, TFAP2C, and SOX17 positive cells in the *BCL2*_OE PGCLCs relative to WT ESC‐derived PGCLCs (Figure 5D). We further validated these results using flow cytometry, which showed a significant decrease in the percentage of cells double‐positive for TNAP and PDPN in *BCL2*_OE (3.66%) compared with wild‐type PGCLCs (15.3%) (Figure 5E).

**FIGURE 5:**
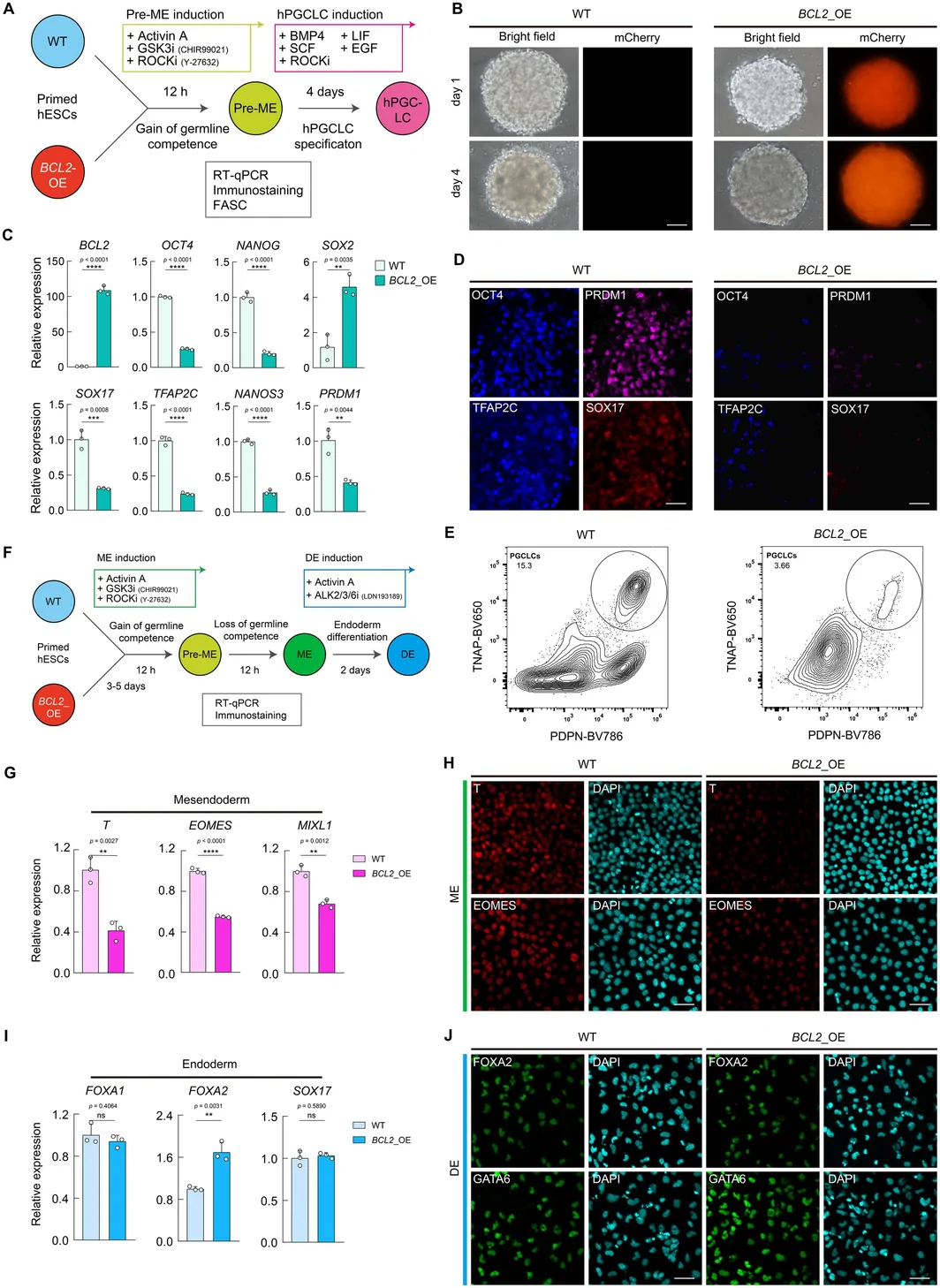
Ectopic *BCL2* expression suppresses mesodermal lineage commitment in human ESCs. (A) Schematic diagram of the PGCLC induction protocol from human primed ESCs. (B) Representative images of embryoid bodies formed from WT and *BCL2*_OE ESCs at day 1 and 4 of differentiation. Scale bar, 100 μm. (C) qPCR analysis of pluripotency genes (*BCL2, OCT4, NANOG, SOX2*) and primordial germ cell markers (*SOX17, TFAP2C, NANOS3, PRDM1*) in WT and *BCL2*_OE ESC‐derived PGCLCs at day 4. (D) Immunostaining of OCT4, PRDM1, TFAP2C, and SOX17 in WT and *BCL2*_OE ESC‐derived PGCLCs at day 4. Scale bar, 50 μm. (E) Flow cytometry analysis of TNAP and PDPN double‐positive cells in WT and *BCL2*_OE ESC‐derived PGCLCs at day 4. (F) Schematic timeline of mesendoderm (ME) and definitive endoderm (DE) differentiation from human primed ESCs. (G) qPCR analysis of early primitive streak markers (*T, EOMES, MIXL1*) in WT and *BCL2*_OE ME cells. (H) Immunostaining of T and EOMES in WT and *BCL2*_OE ME cells. Scale bar, 50 μm. (I) qPCR analysis of definitive endoderm markers (*FOXA1, FOXA2, SOX17*) in WT and *BCL2*_OE DE cells. (J) Immunostaining of FOXA2 and GATA6 protein levels in WT and *BCL2*_OE DE cells. Error bars represent means ± SD (*n* = 3 biological replicates). The *p*‐values were calculated using two‐tailed Student's *t*‐tests (C, G, I), **p < 0.01, ***p < 0.001, ****p < 0.0001, ns: Not significant.

To investigate the mechanism by which *BCL2* inhibits human PGCLCs specification from primed ESCs, we examined the developmental features of competent ESC‐derived differentiation (Figure 5F). Our analysis revealed that *BCL2*_OE ME cells exhibited a significant reduction in early primitive streak (PS) markers *T*, *EOMES*, and *MIXL1* compared to WT ESC‐derived ME (Figure 5G). This downregulation was consistently observed at the protein level via immunostaining for T and EOMES (Figure 5H; Figure S5B). Consistent with this, naïve ESC‐derived *BCL2*_OE ME cells also recapitulated this suppression of PS markers, aligning with the observations in primed ESC‐derived ME cells (Figure S5C,D). Upon differentiation of mesoderm cells into definitive endoderm (DE) over 2 days (Figure 5F), *BCL2* overexpression only slightly or non‐significantly affected the expression of endoderm marker genes (*FOXA1*, *FOXA2*, and *SOX17*) (Figure 5I), with no corresponding significant changes in the protein levels of FOXA2 and GATA6 (Figure 5J). Collectively, our findings indicate that *BCL2* overexpression compromises the differentiation potential of human ESCs, especially for mesendoderm and PGCLC lineage specification.

### 3.6 BCL2 Primes Human ESCs for Dominance in the Mouse ESCs Condition

When mouse and human primed ESCs are co‐cultured under conventional human primed culture conditions, mouse epiblast stem cells (EpiSCs) rapidly become the dominant population, resulting in the swift elimination of human primed ESCs typically within 5 days [43]. *BCL2* overexpression rescues the competitive fitness of human primed ESCs in such interspecies co‐cultures [43]. Under human naïve culture conditions, both human and mouse naïve ESCs are able to proliferate stably in co‐culture systems [43]. However, these co‐culture experiments were conducted in feeder cell‐dependent systems and human ESC‐specific culture media. For the prospective generation of humanized tissues or organs derived from human ESCs, it is therefore essential to overcome the key challenge of adapting human cells to the growth microenvironments of mice or other heterologous species.

We thus hypothesized that *BCL2* overexpression could support the survival and proliferation of human cells under chemically defined mouse ESC culture conditions. To test this, we co‐cultured *BCL2* overexpressing human naïve or primed ESCs with mouse naïve or primed ESCs in feeder‐free conditions, using standard mouse naïve and primed ESCs media, respectively (Figure 6A). Our results showed that under mouse naïve conditions (2i/LIF), human naïve WT ESCs displayed a clear competitive disadvantage, being eliminated within 10 days from an initial 1:1 seeding ratio (Figure 6B). In contrast, human and mouse naïve ESCs co‐proliferated in human naïve culture conditions, as has been previously reported [43]. Strikingly, human naïve *BCL2*_OE ESCs (mCherry) demonstrated a robust ability to co‐proliferate with mouse naïve ESCs (EGFP) under 2i/LIF conditions (Figure 6C).

**FIGURE 6:**
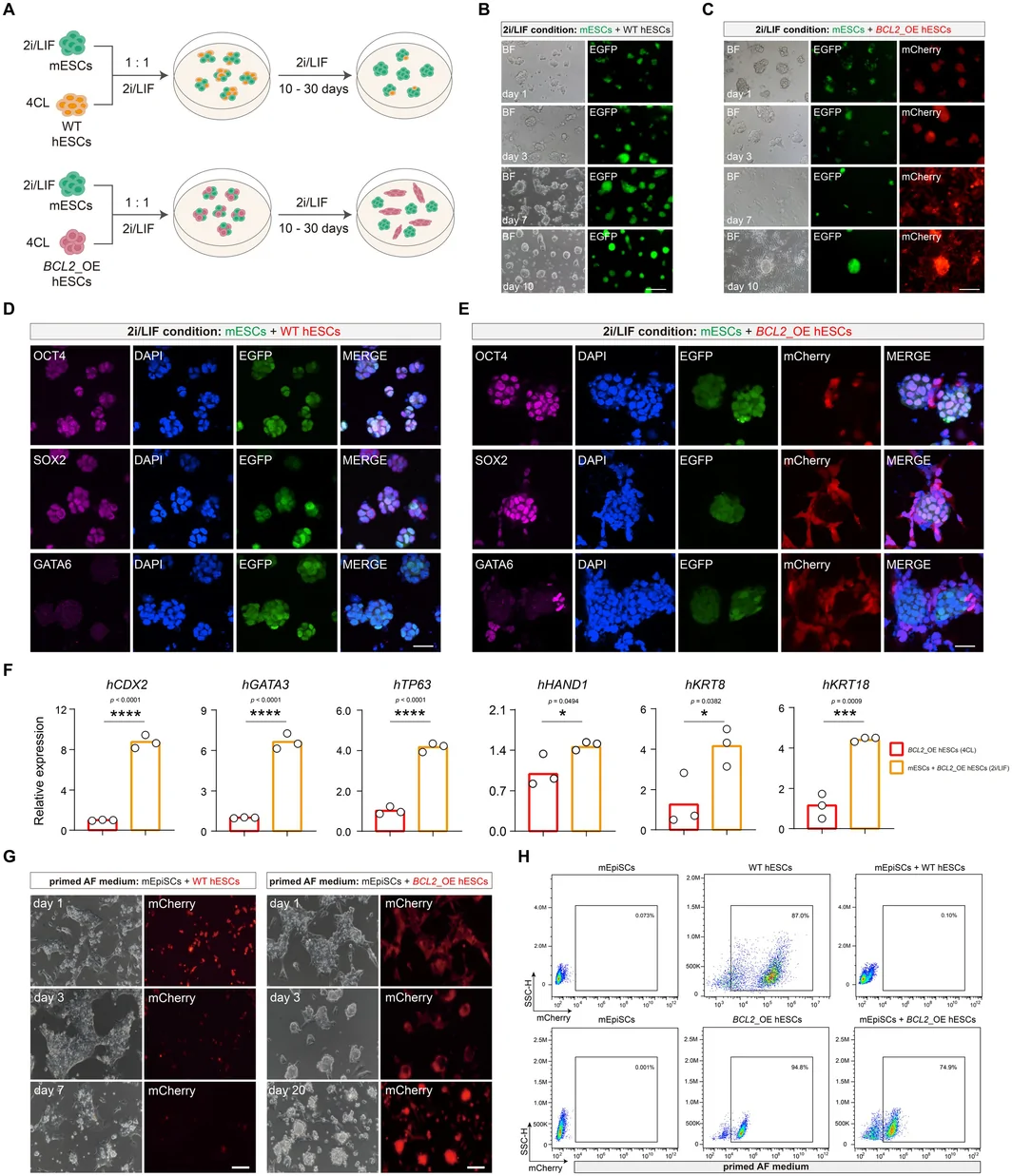
BCL2 primes human ESCs for dominance in the mouse ESCs environment. (A) Schematic diagram of the experimental setup for human naïve ESCs co‐cultured with mouse naïve ESCs under 2i/LIF medium. (B) Representative images of bright field and fluorescence changes in the co‐culture of human naïve WT ESCs with EGFP‐labelled mouse naïve ESCs at day 1, 3, 7, and 10, co‐cultured in 2i/LIF medium. Scale bar, 100 μm. (C) Representative images of bright field and fluorescence changes in co‐culture of mCherry‐labelled human naïve *BCL2*_OE ESCs with EGFP‐labelled mouse naïve ESCs at day 1, 3, 7, and 10, co‐cultured in 2i/LIF medium. Scale bar, 100 μm. (D) Immunostaining of OCT4, SOX2, and GATA6 in human naïve WT ESCs co‐cultured with mouse naïve ESCs at day 7, co‐cultured in 2i/LIF medium. Scale bar, 50 μm. (E) Immunostaining of OCT4, SOX2, and GATA6 in human naïve *BCL2*_OE ESCs co‐cultured with mouse naïve ESCs at day 7, co‐cultured in 2i/LIF medium. Scale bar, 50 μm. (F) qPCR analysis of human trophectoderm genes (*CDX2, GATA3, TP63, HAND1, KRT8, KRT18*) in human naïve *BCL2*_OE ESCs (cultured in 4CL medium) and human naïve *BCL2*_OE ESCs co‐cultured with mouse naïve ESCs at day 7, co‐cultured in 2i/LIF medium. Error bars represent means ± SD (*n* = 3 biological replicates). The *p*‐values were calculated using two‐tailed Student's *t*‐tests, *p < 0.05, ***p < 0.001, ****p < 0.0001, ns: Not significant. (G) Left: Representative images of bright field and fluorescence changes in the co‐culture of human primed WT ESCs (mCherry) with mouse primed EpiSCs at day 1, 3, and 7, cultured in primed AF medium. Scale bar, 100 μm. Right: Representative images of bright field and fluorescence changes in the co‐culture of human primed *BCL2*_OE ESCs (mCherry) with mouse primed EpiSCs at day 1, 3, and 20. Scale bar, 100 μm. (H) Up: Flow cytometry analysis of mouse primed EpiSCs (unlabeled), human primed WT ESCs (mCherry), and their co‐cultured cells at day 20. Below: Flow cytometry analysis of mouse primed EpiSCs (unlabeled), human primed *BCL2*_OE ESCs (mCherry), and their co‐cultured cells at day 20, cultured in primed AF medium.

Next, to assess the pluripotency state of co‐cultured cells under above mentioned conditions, we performed qPCR on samples collected at day 7 of co‐cultures. Our results showed mouse naïve ESCs sustained high pluripotency gene expression levels, regardless of co‐culture with either WT or *BCL2*_OE human naïve ESCs (Figure S6A,B). In contrast, human naïve ESCs displayed pronounced downregulation of pluripotency genes, a trend that was even observed in the *BCL2*_OE ESCs (Figure S6C,D). This observation was further confirmed by immunostaining (Figure 6D,E). Thus, BCL2 confers upon human ESCs the ability to compete and survive in the mouse 2i/LIF environment, albeit at the cost of diminished pluripotency.

To further characterize the resultant cellular state, we analyzed the expression profiles of lineage specification markers. This analysis revealed that *BCL2*_OE ESCs acquired a distinct transcriptional bias toward the trophoblast lineage, as evidenced by elevated expression of trophoblast markers (Figure 6F), suppressed expression of mesodermal genes (Figure S6E), and variable expression of hypoblast‐associated genes (Figure S6F). Importantly, human naïve *BCL2*_OE ESCs co‐cultured with mouse naïve ESCs under 2i/LIF conditions maintained at a detectable frequency (12.7%) even after 26 days co‐cultures (Figure S6G,H). In addition, we co‐cultured *BCL2* overexpressing human primed ESCs (cultured in mTeSR) with mouse primed EpiSCs under feeder‐free conditions using standard mouse primed EpiSC medium (N2B27 supplemented with ACTIVIN A and bFGF). We found that human primed WT ESCs (mCherry) became nearly undetectable within 7 days of co‐culture (Figure 6G). By contrast, human primed *BCL2*_OE ESCs (mCherry) gained a competitive advantage and evolved into the dominant population, making up 74.9% of the total cell population after 20 days of co‐culture (Figure 6H). This result demonstrates that human *BCL2*_OE ESCs gain a competitive over mouse ESCs under mouse‐adapted culture conditions, even upon exit from pluripotency.

## 4 Discussion

In this study, we establish BCL2 as a regulator that both stabilizes human naïve pluripotency and facilitates the acquisition and maintenance of an 8C‐like state. Our findings reveal a multifaceted role for BCL2, positioning it as a key node linking pluripotency maintenance, lineage priming, and cellular competitiveness (Figure 7). These insights offer a useful model for investigating 8C‐like state activation in human embryonic stem cells. The 8C‐like state in human ESCs recapitulates key features of zygotic genome activation (ZGA), a pivotal event in early embryonic development that has drawn considerable interest across stem cell biology, development, and gene regulation [44, 45, 46]. In human embryos, the transcriptional burst at the 8‐cell stage is tightly associated with the acquisition of totipotency—a transient developmental window that is subsequently silenced as embryogenesis proceeds [47]. Although human naïve ESCs can spontaneously adopt an 8‐cell‐like cell (8CLC) state that recapitulates key features of this totipotent phase, the regulatory mechanisms that drive this reprogramming process remain poorly defined [48]. Here, we identify BCL2 as a regulator that not only maintains human naïve pluripotency but also establishes and stabilizes the 8C‐like state. Our findings suggest the involvement of BCL2 in cell fate determination, facilitating the transition from pluripotency to an 8C‐like state in human naïve ESCs. Importantly, the effects of *BCL2* overexpression on pluripotency maintenance and 8C‐like state activation were further validated in an independent human chemically induced pluripotent stem cell (CiPSC) line (Figure S6I–M). These results reinforce that the regulatory role of BCL2 in naïve pluripotency and 8CLC stabilization is not restricted to the H9 background, thereby supporting the broader generalizability of our conclusions. While our results already reveal the intrinsic heterogeneity of the 8C‐like state, we acknowledge that single‐cell resolution would provide further insights into this feature. The observed enhancement of naïve‐state stability, 8C‐like state activation, and cellular competitiveness in *BCL2*‐overexpressing ESCs may therefore be at least partially attributable to improved cell survival via its canonical anti‐apoptotic function. Nevertheless, we cannot exclude the possibility that BCL2 also regulates naïve or 8CLC‐like identity through direct mechanisms or alternative pathways, through its classical role in anti‐apoptosis. In addition, our results suggest that BCL2 may maintain the pluripotency of human embryonic stem cells by improving cellular state through its anti‐apoptotic function, which consequently contributes to H3K27ac enrichment at core pluripotency gene loci. Therefore, BCL2 likely exerts an indirect effect on H3K27ac rather than directly participating in this histone modification. Further studies are warranted to dissect the relative contributions of survival‐dependent and state‐specific effects.

**FIGURE 7:**
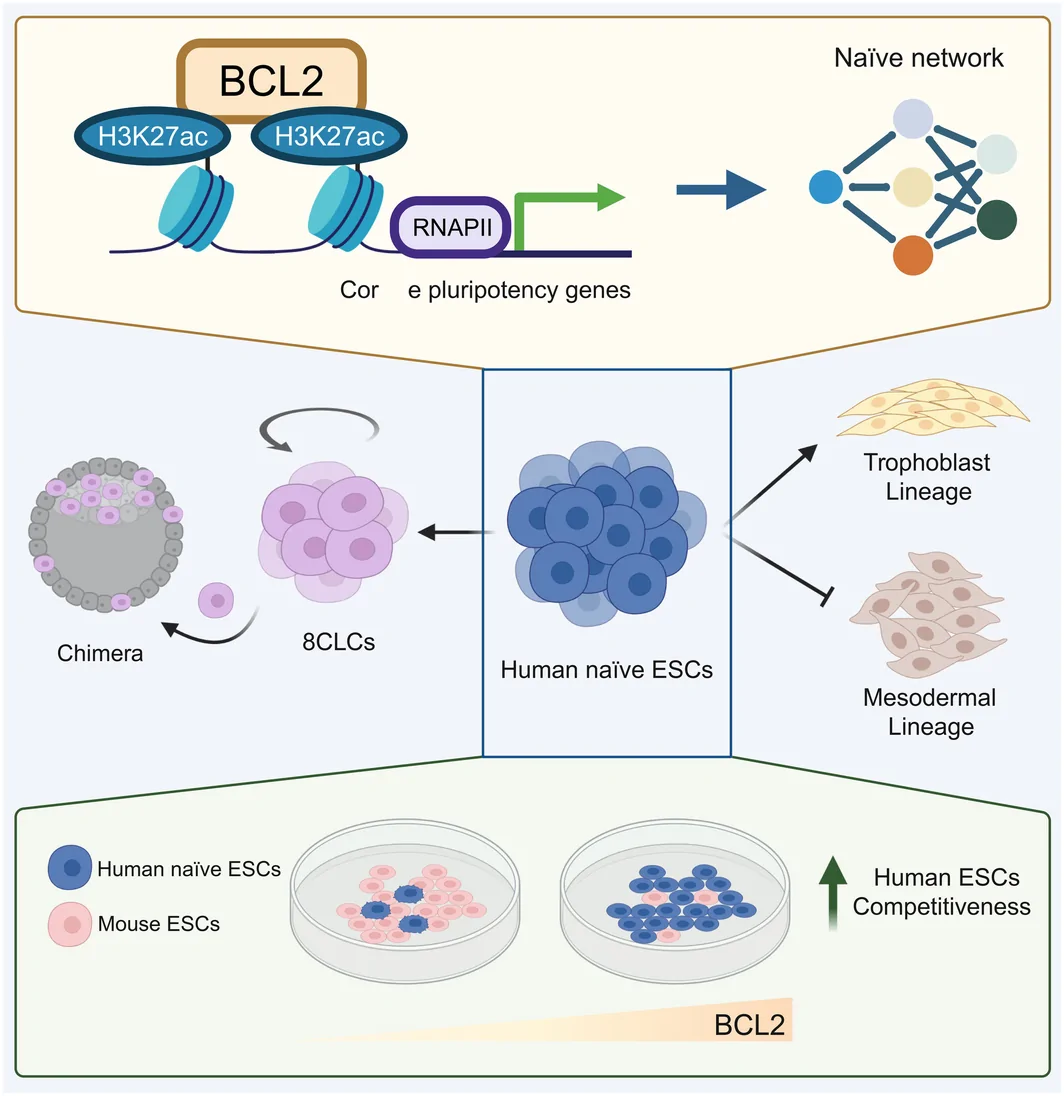
Schematic showing the working model of BCL2 in regulating the 8CLC program and pluripotency. Our study positions BCL2 as a regulator that anchors naïve pluripotency and enables a stable 8C‐like fate. We demonstrate that BCL2 regulates pluripotency and induces an 8C‐like state, linking pluripotency maintenance, lineage priming, and cellular competitiveness.

During early embryonic development, mice and humans exhibit distinct expression dynamics of core pluripotency genes [49, 50, 51, 52], a difference that is recapitulated in their respective in vitro 2CLC and 8CLC models [4, 5, 53]. Previous studies have shown that induction of mouse 2‐cell‐like cells (2CLCs) is typically accompanied by downregulation of pluripotency networks. Notably, although pluripotency gene expression is reduced, 2CLCs exclusively emerge within ESC populations as a pluripotency‐specific cell state and are not detected in non‐pluripotent cell types, a process largely dependent on core regulators such as DUX and its target genes [54, 55, 56, 57]. In contrast, the activation of human 8CLCs reveals a more complex regulatory pattern, although certain pluripotency factors (e.g., *SOX2*) may show a declining trend in some studies, most core pluripotency genes remain expressed in 8CLCs and can even be co‐expressed with 8CLC‐specific markers [4, 5, 27]. This species‐specific divergence likely reflects fundamental differences in regulation and the molecular mechanisms that stabilize the 2C‐like and the 8C‐like state activation [58, 59, 60].

Our data support a model wherein BCL2 functions as a molecular bridge connecting naïve pluripotency with the 8C‐like state through maintenance of core pluripotency factors. Although our current study does not establish a direct causal link between BCL2 and H3K27ac‐mediated epigenetic regulation, it does reveal a consistent association between *BCL2* overexpression and H3K27ac enrichment at key pluripotency and lineage‐biased gene loci, providing a foundation for future mechanistic dissection. Importantly, our results reveal a striking species‐specific difference; while BCL2 is readily detectable in mouse ESCs [30, 61, 62], its endogenous expression is almost undetectable in human ESCs. Conversely, *BCL2* overexpression in human ESCs supports naïve pluripotency and the 8C‐like state activation, leading to a unique transcriptional and epigenetic response that is not fully recapitulated in mouse models. The sustained expression of pluripotency factors in *BCL2*‐overexpressing 8CLCs may provide a critical foundation for maintaining self‐renewal capacity while retaining lineage‐biased differentiation potential of human embryonic stem cells. However, our current data cannot distinguish whether BCL2 increases the proportion of 8CLC‐positive cells or simply upregulates marker expression within pre‐existing subpopulations. Future single‐cell‐resolution studies will be required to quantify 8CLC‐positive cells and dissect cellular heterogeneity upon *BCL2* overexpression.

The functional significance of this regulatory program is demonstrated by the remarkable transcriptional resemblance of BCL2‐induced 8CLCs. Their ability to contribute to both embryonic and extra‐embryonic lineages in chimeric embryos provides compelling evidence of lineage‐biased differentiation capacity. Previous studies have shown that *BCL2* overexpression enhances the competitive fitness of human ESCs in co‐culture systems under conventional human ESC culture conditions [30]. Extending these findings, we reveal that BCL2 not only supports human ESCs' proliferation in mouse ESCs culture conditions but also confers a surprising competitive advantage over their mouse counterparts. This interspecies dominance, together with the observed suppression of mesodermal commitment and a pronounced bias toward trophoblast differentiation, suggests that BCL2 not only induces 8C‐like state but also actively primes specific lineage choices. However, we cannot rule out the possibility that the TE contribution of *BCL2*‐overexpressing cells compared with controls in mouse chimera assays may result from prolonged e4CL culture or a survival advantage conferred by *BCL2* overexpression.

The molecular framework established here provides a foundation for future studies exploring how BCL2 interfaces with other known regulators of totipotency and how its activity is normally constrained during developmental progression. Notably, since the initial discovery of 8C‐like cells, most studies have focused primarily on optimizing in vitro culture conditions to induce 8C‐like cells [5, 27], TBLCs [14], and other related cell states [63]. We have recently reported the regulatory role of NELFA in activating 8C‐like cells in human naïve ESCs [64], yet the identification of additional genes that regulate this process remains unclear. Thus, our study suggests a positive role for BCL2 in this process, representing a key finding of this work. In conclusion, our work demonstrates that BCL2 contributes to the establishment of an 8C‐like state in human naïve ESCs. The stable, self‐renewing 8C‐like state established by *BCL2* overexpression provides a powerful platform for dissecting the mechanisms governing human zygotic genome activation, totipotency, and lineage specification. This system provides a valuable platform for studying the principles of 8C‐like cell induction in vitro and may offer insights into the controlled manipulation of stem cell plasticity.

## 5 Conclusion

Our findings show that *BCL2* overexpression helps maintain human naïve pluripotency and promotes the induction and long‐term culture of 8C‐like cells. *BCL2*‐overexpressing 8C‐like cells retain core pluripotency gene expression, contribute to both embryonic and extra‐embryonic lineages, and outcompete mouse ESCs in mouse culture conditions. This study provides a useful in vitro system for further exploring the regulatory mechanisms of 8C‐like state activation and human zygotic genome activation.

## Author Contributions

B.W. initiated and led the project, conceived, designed, and supervised experiments, analysed results and wrote the manuscript. Z.L., H.W., X.S., F.Y. and B.W. performed most experiment. S.B. and X.L. provided technical assistance. B.W. and H.Y. analysed figures and wrote the manuscript with inputs from all authors.

## Funding

This study is supported by the Inner Mongolia Natural Science Foundation Project for Outstanding Young Scholars (2025JQ020 to B.W. and H.Y.), the National Natural Science Foundation of China (32460837 to B.W.), the Major Science and Technology Projects of Inner Mongolia Autonomous Region (2026ZD0079 to B.W.), the National Science and Technology Major Project of China (2023ZD0405001 to B.W.), the Program for Young Talents of Science, the Technology in Universities of Inner Mongolia Autonomous Region (NJYT23091 to B.W.), the National Key Research and Development Program of China (2022YFD1302202 to S.B.) and the Program of “JIE BANG GUA SHUAI” of Inner Mongolia (2022JBGS0021 to X.L.).

## Ethics Statement

All experiments were conducted in accordance with the relevant guidelines and regulations for human embryonic stem cell research and in compliance with the ISSCR 2021 guidelines [65]. All experiments involving human embryonic stem cells and human‐mouse ex vivo interspecies chimeric assays were reviewed and approved by the Bioethics Committee of Inner Mongolia University (EC‐(2023)‐0316). Mouse handling and experiments were carried out with humane methods in compliance with the guidelines of the Institutional Animal Care and Use Committee, Inner Mongolia University (SYXK(MENG)2020‐0006). Together, this investigation falls within the existing legislation set by regulatory bodies in China. This study did not involve the use of human preimplantation embryos.

## Consent

The authors have nothing to report.

## Conflicts of Interest

The authors declare no conflicts of interest.

## Supporting information


**Figure S1:** cpr70297‐sup‐0001‐Figure_S1‐S6.pdf. *BCL2* overexpression does not impair pluripotency in human ESCs. (A) Schematic of piggyBac transposon system for generating stable *BCL2*‐overexpressing human primed ESCs. (B) Representative morphology of WT (p38) and *BCL2*_OE (p14) primed ESCs. Scale bar: 100 μm. (C) qPCR analysis of *BCL2* and pluripotency genes (*OCT4*, *SOX2*, *NANOG*) in WT (p38) and *BCL2*_OE (p14) primed ESCs. (D) Western blot analysis of BCL2 protein levels in WT (p38) and *BCL2*_OE (p14) ESCs. (E, F) Immunostaining of OCT4, NANOG, and SOX2 in WT (p38) (E) and *BCL2*_OE (p14) (F) primed ESCs. Scale bar, 50 μm. (G) Volcano plot of differentially expressed genes in *BCL2*_OE versus WT primed ESCs. (H) Distribution of expression changes for 587 primed pluripotency‐associated genes in *BCL2*_OE versus WT ESCs. (I) Heatmap showing expression patterns of selected pluripotency‐related genes in WT and *BCL2*_OE primed ESCs. (J) GSEA of biological processes enriched in *BCL2*_OE versus WT primed ESCs. (K) Western blot quantification analysis of OCT4, NANOG, and SOX2 protein levels in WT and *BCL2*_OE ESCs of Figure 1E. (L) Volcano plot of differentially expressed genes in *BCL2*_OE versus WT ESCs cultured in 4CL medium. (M) Expression profile of 124 known naïve genes in *BCL2*_OE versus WT ESCs cultured in 4CL medium. (N) RNA‐seq read counts of *BCL2* in control and *BCL2*‐overexpressing (*BCL2*_OE) ESCs. *BCL2* reads were barely detectable in control cells (range 40–70) but markedly elevated upon overexpression (range 1300–1600). (O) Immunostaining of BCL2 (green) in WT and *BCL2*_OE ESCs. Nuclei were counterstained with DAPI (blue). Scale bar, 50 μm. (P) Schematic illustration of the inducible CRISPRi system used to target *BCL2*. (Q) qPCR analysis of *BCL2* mRNA expression in WT and *BCL2*‐targeting CRISPRi cells following 5 days induction. (R) Western blot analysis of BCL2 protein levels in control and *BCL2*‐targeting CRISPRi cells following 5 days induction. β‐ACTIN was used as a loading control. Error bars represent means ± SD (*n* = 3 biological replicates). The *p*‐values were calculated using two‐tailed Student's *t*‐tests (C, K, Q), *p < 0.05, **p < 0.01, ***p < 0.001, ****p < 0.0001, ns: not significant.
**Figure S2:** Molecular characterization of BCL2‐induced 8C‐like state. (A) Immunostaining of key pluripotency proteins (OCT4, SOX2, NANOG) in WT ESCs following 6‐day e4CL induction. Scale bar, 50 μm. (B) Relative RNA expression levels of key naïve pluripotency genes (*DPPA3, KLF4, DPPA5, SALL4, TFCP2L1*) in WT and *BCL2*_OE ESCs after 6‐day e4CL induction. Error bars represent means ± SD (*n* = 3 biological replicates). The *p*‐values were calculated using two‐tailed Student's *t*‐tests, *p < 0.05, **p < 0.01, ***p < 0.001, ****p < 0.0001, ns: not significant. (C) Immunostaining of TPRX1 and ZSCAN4 in WT ESCs after 6‐day e4CL induction. Scale bar, 50 μm. (D) Volcano plot displaying differentially expressed genes in *BCL2*_OE versus WT ESCs after 6‐day e4CL induction.
**Figure S3:** Molecular characterization of long‐term 8C‐like culture. (A) Morphology of long‐term culture of WT and *BCL2*_OE ESCs under e4CL conditions. Scale bar, 100 μm. (B) Karyotyping of e4CL‐*BCL2*_OE 8CLCs at passages 10. (C) Immunostaining validation of 8C‐like markers (TPRX1, ZSCAN4) in WT and *BCL2*_OE ESCs under 18‐day e4CL medium. Scale bar, 50 μm. (D) Quantification of western blot results from three independent experiments for proteins shown in Figure 3D. Error bars represent means ± SD (*n* = 3 biological replicates). The *p*‐values were calculated using two‐tailed Student's *t*‐tests, *p < 0.05, **p < 0.01, ***p < 0.001, ****p < 0.0001, ns: not significant. (E) Volcano plot showing distribution of differentially expressed genes between *BCL2*_OE and WT ESCs after 18‐day e4CL culture. (F) Comparative overlap analysis showing significantly enrichment of *BCL2*‐upregulated and downregulated genes in published human day 3 embryo and 8C‐like gene sets. (G) Gene set enrichment analysis (GSEA) of biological processes enriched in *BCL2*_OE ESCs compared to WT ESCs after 18‐day e4CL culture.
**Figure S4:** Dissecting the regulatory mechanism and functional properties of BCL2‐induced 8C‐like cells. (A) H3K27ac CUT&Tag peaks at promoter and gene body regions of core pluripotency genes (*BCL2L1, TFAP2C, DNMT3L, KLF5, BMP4, JUP*) in e4CL‐*BCL2*_OE ESCs (p12) cultured in e4CL medium compared to 4CL‐WT ESCs (p28) and 4CL‐*BCL2*_OE ESCs (p30). (B) Additional motif enrichment analysis results for differential H3K27ac peaks in *BCL2*_OE ESCs cultured in e4CL medium. (C) Gene set enrichment analysis (GSEA) of transcriptomic data from e4CL‐*BCL2*_OE ESCs upregulated genes, showing enrichment for cell adhesion, JAK–STAT signalling, and WNT signalling pathways compared to the same 18‐day e4CL cultured e4CL‐WT ESCs. (D) H3K27ac CUT&Tag peaks at promoter and gene body regions of core TE‐like genes (*KRT18, RARG*) in *BCL2*_OE ESCs cultured in e4CL medium compared to 4CL‐WT ESCs and 4CL‐*BCL2*_OE ESCs. (E) Additional motif enrichment analysis results for differential H3K27ac peaks in *BCL2*_OE ESCs cultured in e4CL medium. (F) Transcriptional levels of trophectoderm‐related genes in e4CL‐*BCL2*_OE versus e4CL‐WT ESCs under 18‐day e4CL induction. (G) Immunostaining of ELF5 and OCT4 in WT and *BCL2*_OE ESC‐derived TS‐like cells. Scale bar, 50 μm. (H) Representative images chimeric blastocyst immunostaining using OCT4 and CDX2 antibodies. Eight‐cell stage embryos were injected with mCherry‐labelled e4CL‐WT ESCs and then cultured for 48–60 h in vitro. The cell nuclei were stained with DAPI. Scale bars, 50 μm.
**Figure S5:** Ectopic BCL2 expression suppresses the mesodermal lineage commitment of human ESCs. (A) Representative images of embryoid bodies formed from WT and *BCL2*_OE ESCs at day 2 and 3 of differentiation. Scale bar, 100 μm. (B) Immunostaining for T and EOMES in WT and *BCL2*_OE Pre‐ME cells. (C) qPCR analysis of early PS markers (*T*, *EOMES*, *MIXL1*) in naïve ESC‐derived (4CL) WT and *BCL2*_OE Pre‐ME and ME cells. (D) qPCR analysis of late PS markers (*TBX6*, *MESP1*, *MESP2*) in naïve ESC‐derived (4CL) WT and *BCL2*_OE Pre‐ME and ME cells. Error bars represent means ± SD (*n* = 3 biological replicates). The *p*‐values were calculated using two‐tailed Student's *t*‐tests (C, D), *p < 0.05, **p < 0.01, ***p < 0.001, ****p < 0.0001, ns: not significant.
**Figure S6:** BCL2 primes human ESCs for dominance in the mouse ESCs environment. (A) qPCR analysis of mouse pluripotency genes (*Oct4, Sox2, Nanog*) in mouse naïve ESCs and mouse naïve ESCs co‐cultured with human naïve WT ESCs at day 7 in 2i/LIF medium. (B) qPCR analysis of mouse pluripotency genes (*Oct4, Sox2, Nanog*) in mouse naïve ESCs and mouse naïve ESCs co‐cultured with human naïve *BCL2*_OE ESCs at day 7 in 2i/LIF medium. (C) qPCR analysis of human pluripotency genes (*OCT4, SOX2, NANOG*) in human naïve WT ESCs (4CL) and human naïve WT ESCs co‐cultured with mouse naïve ESCs at day 7 in 2i/LIF medium. (D) qPCR analysis of human pluripotency genes (*OCT4, SOX2, NANOG*) in human naïve *BCL2*_OE ESCs and human naïve *BCL2*_OE ESCs (4CL) co‐cultured with mouse naïve ESCs at day 7 in 2i/LIF medium. (E) qPCR analysis of human mesoderm genes (*MESP1, MESP2, MIXL1, TBXT*) in human naïve *BCL2*_OE ESCs (4CL) and human naïve *BCL2*_OE ESCs co‐cultured with mouse naïve ESCs at day 7 in 2i/LIF medium. (F) qPCR analysis of human hypoblast genes (*FOXA1, GATA4, GATA6*) in human naïve *BCL2*_OE ESCs (4CL) and human naïve *BCL2*_OE ESCs co‐cultured with mouse naïve ESCs at day 7 in 2i/LIF medium. (G) Flow cytometry analysis of human naïve WT ESCs (unlabeled), mouse naïve ESCs (EGFP), and their co‐cultured cells at day 26 in 2i/LIF medium. (H) Flow cytometry analysis of mouse naïve ESCs (EGFP), human naïve *BCL2*_OE ESCs (mCherry), and their co‐cultured cells at day 26 in 2i/LIF medium. (I,J) Morphological observation (I) and qPCR analysis of core pluripotency marker expression (J) in wild‐type (WT) and *BCL2*‐overexpressing (*BCL2*_OE) CiPSCs cultured under standard mTeSR conditions. No obvious morphological differences or significant changes in pluripotency gene expression were observed between the two cell groups. (K, L) Representative cell morphology (K) and qPCR quantification of pluripotency‐associated gene levels (L) in WT and *BCL2*_OE CiPSCs after 4CL treatment. Consistent with the phenotypes in H9 ESCs, *BCL2* overexpression markedly upregulated the expression of pluripotency genes in CiPSCs under the 4CL condition. (M) qPCR analysis of 8C‐like marker genes (including *ZSCAN4, DUXA, H3.Y/X,* and *LEUTX*) in *BCL2*_OE CiPSCs cultured under 4CL and e4CL conditions. *BCL2*OE CiPSCs exhibited robust activation of 8C‐like genes upon e4CL treatment, while WT CiPSCs failed to survive under the e4CL condition. Error bars represent means ± SD (*n* = 3 biological replicates). The *p*‐values were calculated using two‐tailed Student's *t*‐tests (A‐F, J, L, M), *p < 0.05, **p < 0.01, ***p < 0.001, ****p < 0.0001, ns: not significant.

**Supplementary Table 1:** DEG in mTeSR_WT versus mTeSR_BCL2_OE. Related to Figure S1G.
**Supplementary Table 2:** FPKM of the selected genes. Related to Figure S1H.
**Supplementary Table 3:** DEG in 4CL_WT versus 4CL_BCL2_OE. Related to Figure S1L.
**Supplementary Table 4:** FPKM of different samples. Related to Figure 1F,G.
**Supplementary Table 5:** FPKM of the selected genes. Related to Figure S1M.
**Supplementary Table 6:** DEG in e4cl_day6_WT versus e4cl_day6_BCL2_OE. Related to Figure S2D.
**Supplementary Table 7:** Venn gene lists. Related to Figure 2I.
**Supplementary Table 8:** FPKM of different samples. Related to Figure 2J.
**Supplementary Table 9:** FPKM of different samples. Related to Figure 3E,H.
**Supplementary Table 10:** DEG in e4cl_day18_WT versus e4cl_day18_BCL2_OE. Related to Figure S3E.
**Supplementary Table 11:** FPKM of human E3 genes and 8C‐like genes in different samples. Related to Figure 3G.
**Supplementary Table 12:** Venn gene lists. Related to Figure S3F.
**Supplementary Table 13:** RT‐qPCR Primers and CRISPRa sgRNA Sequences.
**Supplementary Table 14:** The uncropped western blots.

## Data Availability

RNA‐seq and CUT&Tag‐seq datasets are available on Genome Sequence Archive for Human under GSA‐Human Accession number HRA015308. All other data supporting the findings of this study are available from the corresponding authors on reasonable request.
