# Supplementary material for: BCL2 Facilitates Naïve Pluripotency and an 8C‐Like State in Human Embryonic Stem Cells
Source: Cell Prolif. 2026 Nov 8:e70297. Online ahead of print. doi: 10.1111/cpr.70297 (PMC13744942; doi:10.1111/cpr.70297)

Figure S1. *BCL2* overexpression does not impair pluripotency in human ESCs

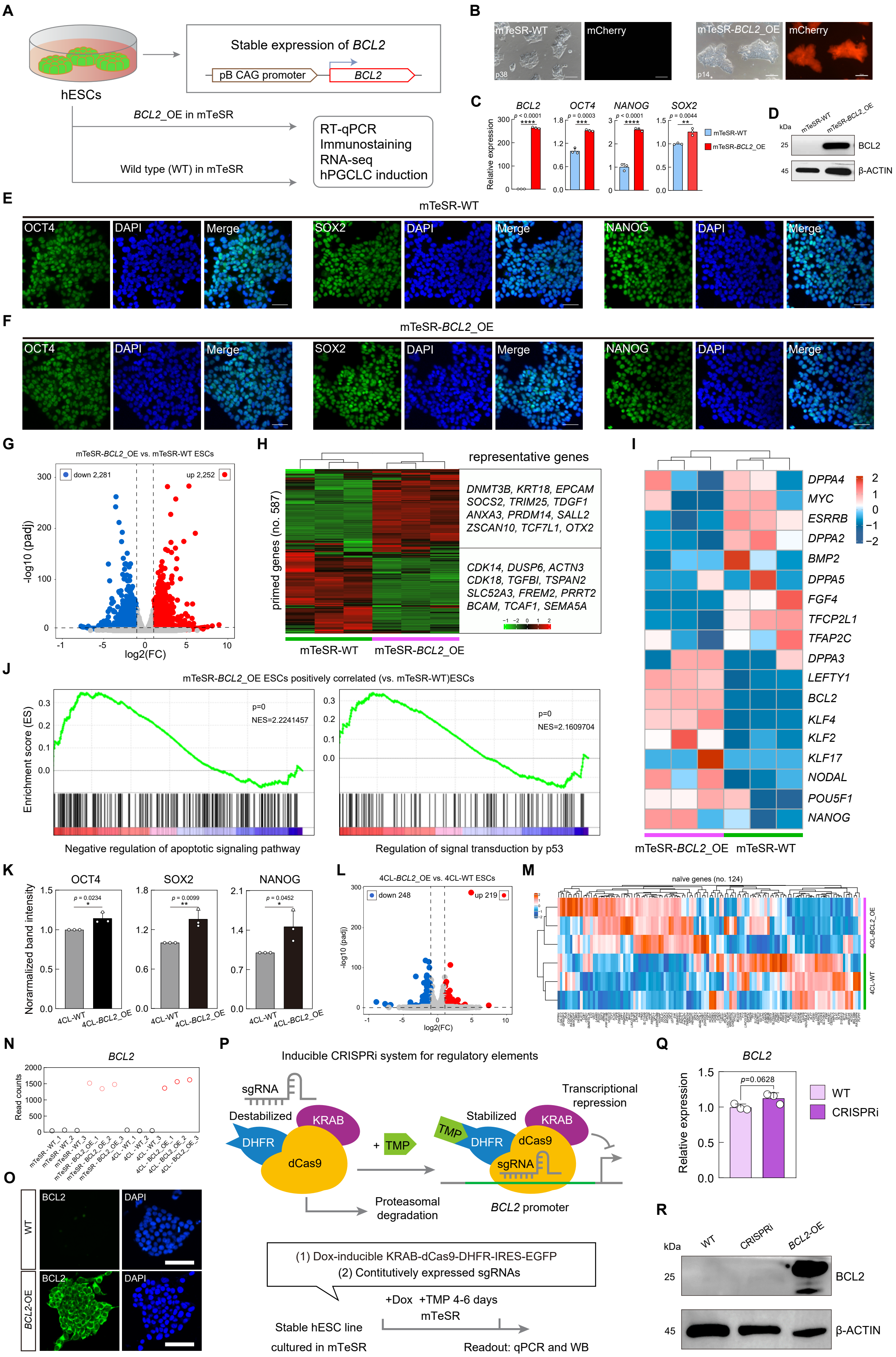

Figure S2. Molecular characterization of BCL2-induced 8C-like state

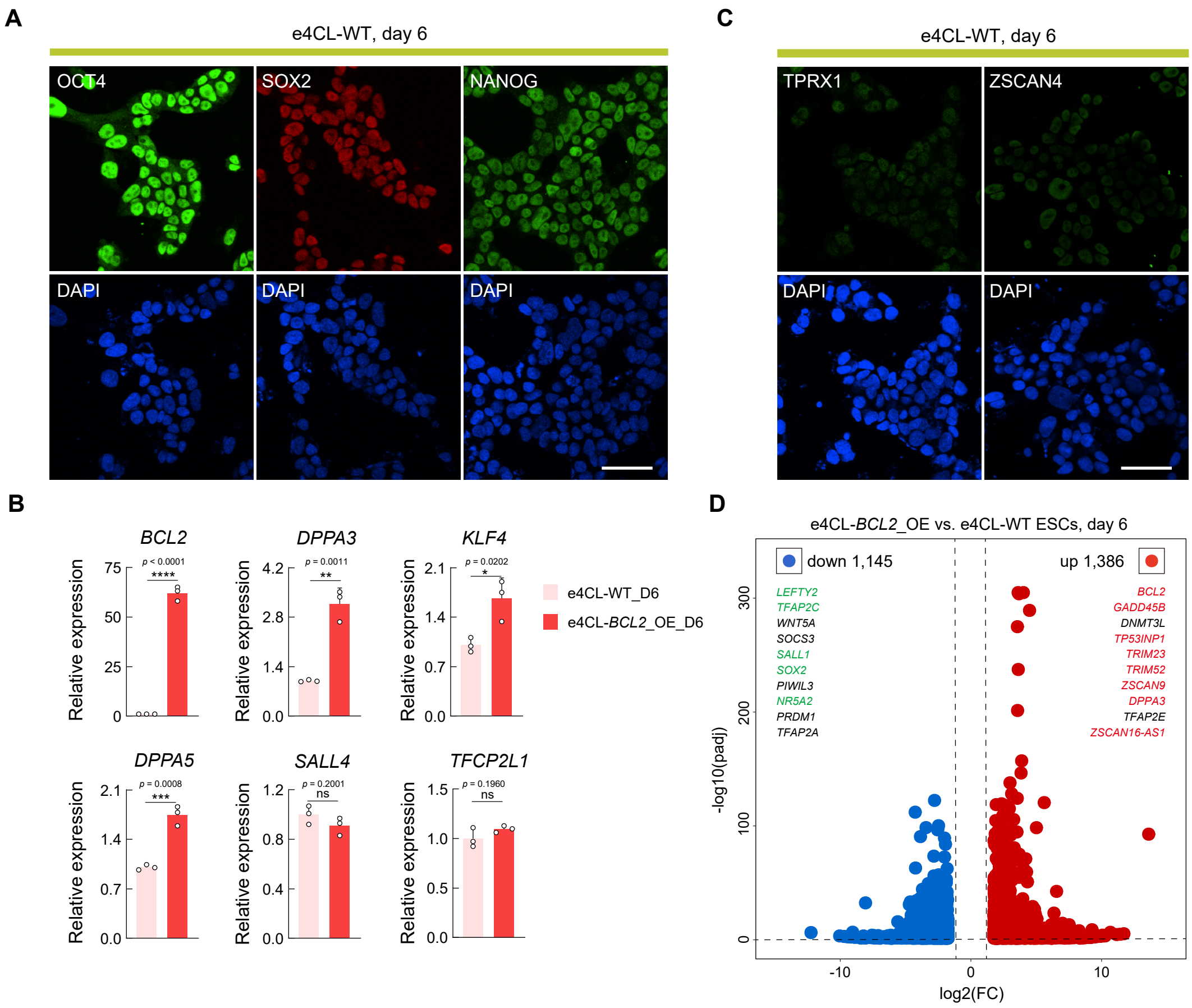

Figure S3. Molecular characterization of long-term 8C-like culture

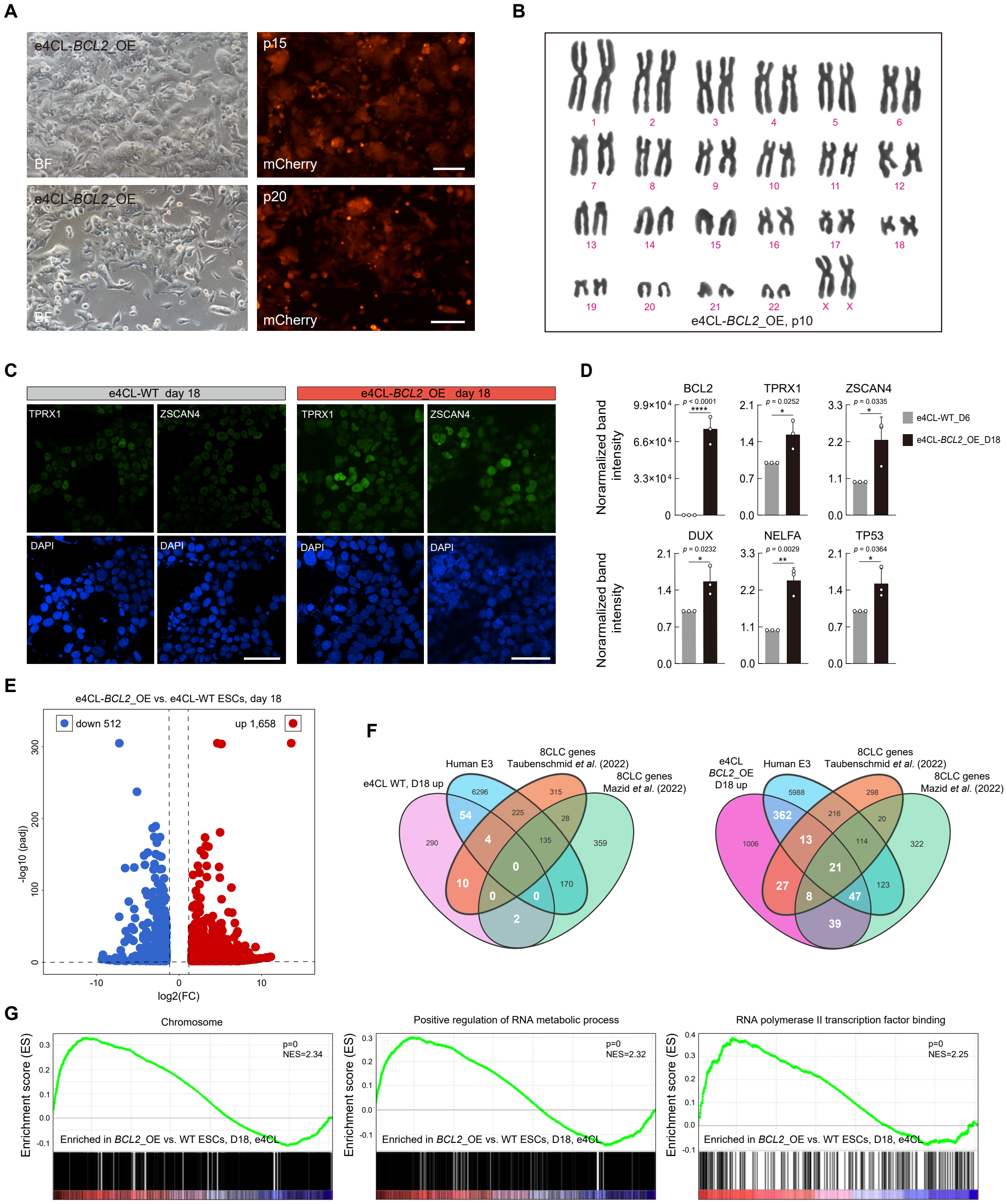

Figure S4. Dissecting the regulatory mechanism and functional properties of 8-cell like cells

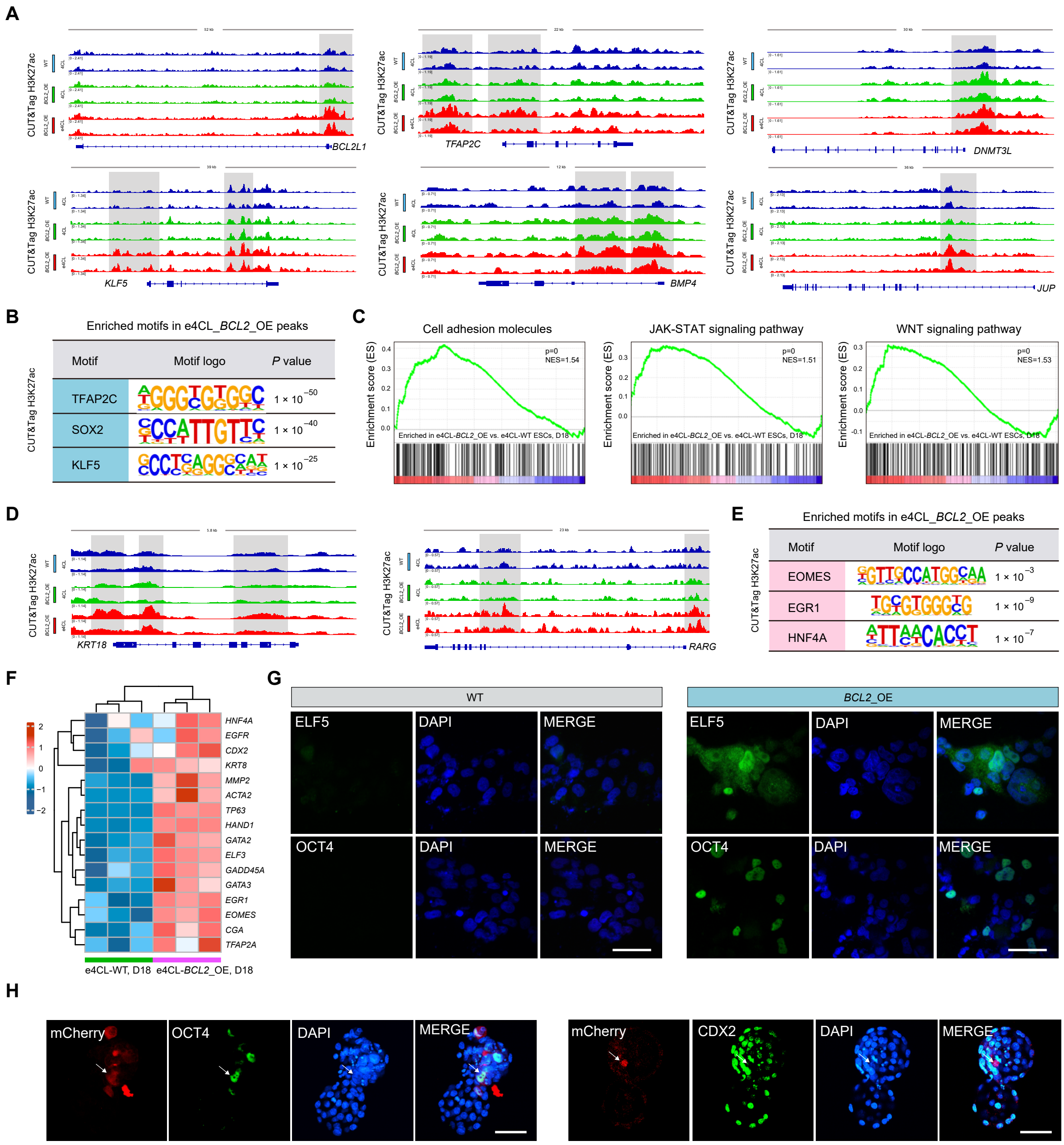

Figure S5. Ectopic BCL2 expression suppresses the mesodermal lineage commitment of human ESCs

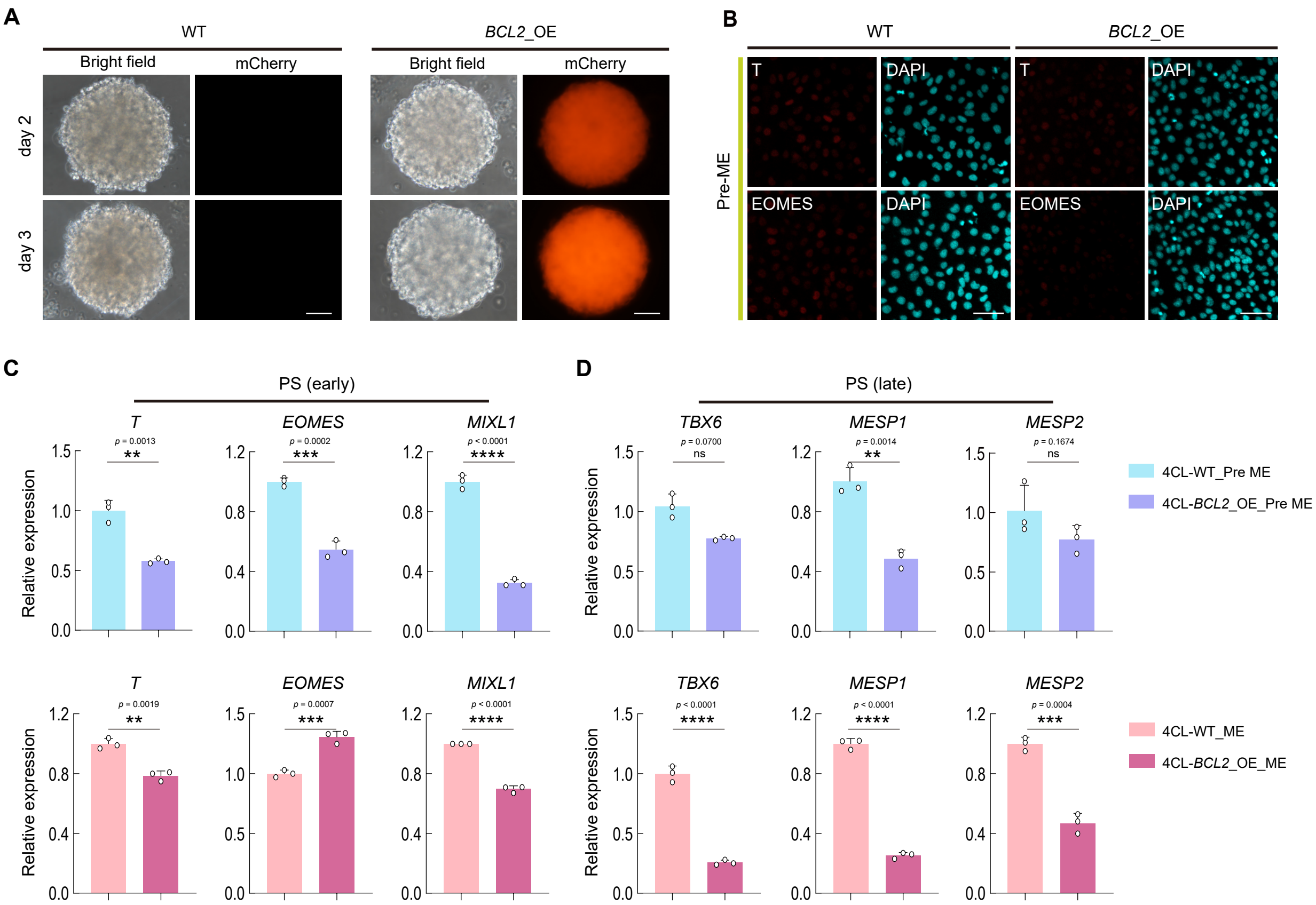

Figure S6. BCL2 primes human ESCs for dominance in the mouse ESCs environment

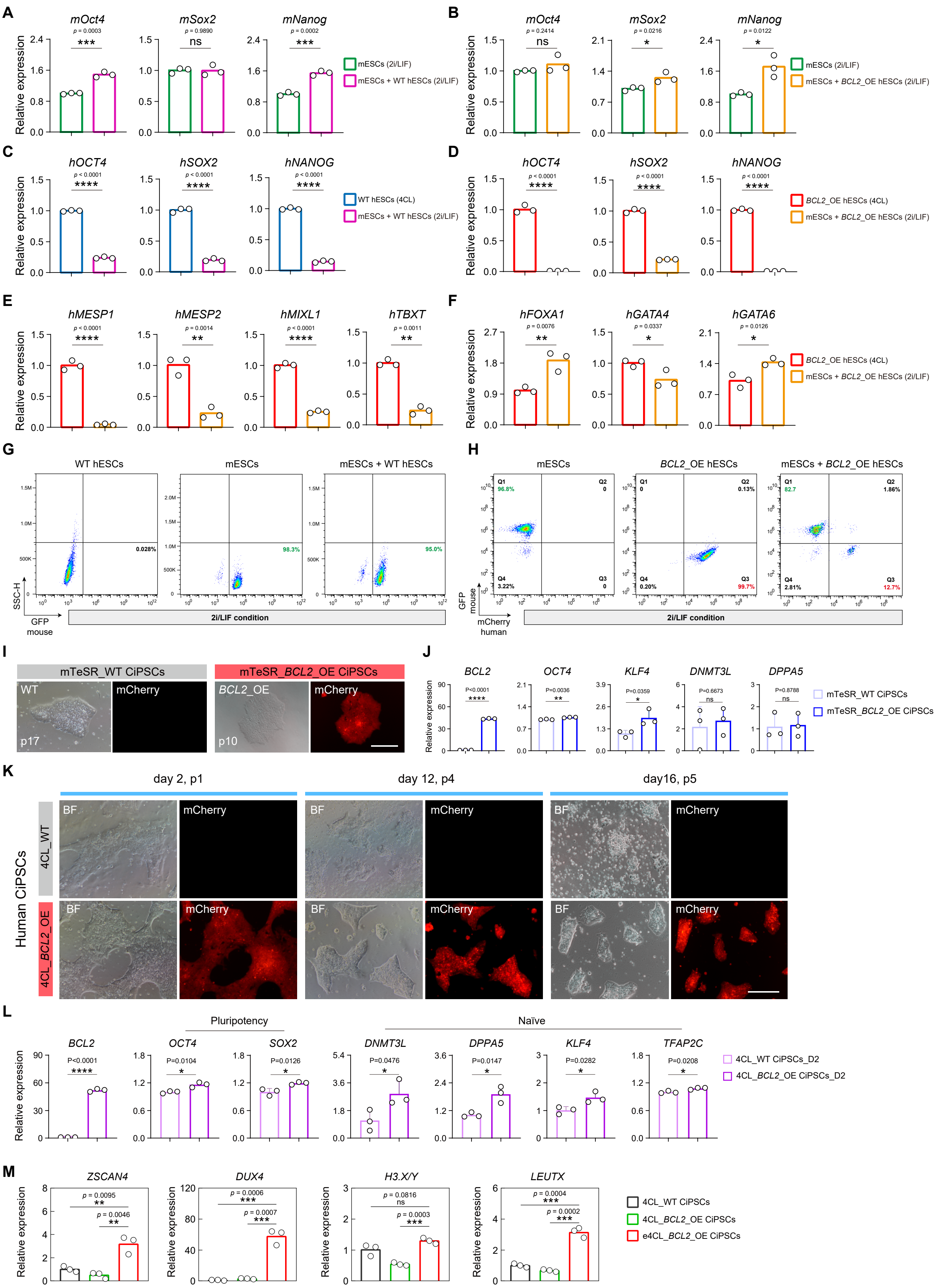

Supplement: Supplementary file 1 [file CPR-9999-e70297-s002.pdf]
